# Photochemically Assisted Synthesis of Thienobenzotriazole-Based Dual Cholinesterase Inhibitors

**DOI:** 10.3390/molecules30163439

**Published:** 2025-08-20

**Authors:** Antonija Jelčić, Stanislava Talić, Ilijana Odak, Paula Pongrac, Dora Štefok, Irena Škorić

**Affiliations:** 1 Department of Organic Chemistry, University of Zagreb Faculty of Chemical Engineering and Technology, Trg Marka Marulića 19, HR-10 000 Zagreb, Croatia; ajelcic@fkit.unizg.hr; 2 Department of Chemistry, Faculty of Science and Education, University of Mostar, Matice Hrvatske bb, 88 000 Mostar, Bosnia and Herzegovina; ilijana.odak@fpmoz.sum.ba; 3 Faculty of Biotechnology and Drug Development, University of Rijeka, Radmile Matejčić 2, HR-51 000 Rijeka, Croatia; paula.pongrac@selvita.com (P.P.); dora.stefok@selvita.com (D.Š.)

**Keywords:** anti-inflammatory activity, cholinesterase, non-selective inhibition, thienobenzo-1,2,3-triazolinium salts

## Abstract

Background: It has been shown previously that thienobenzo-1,2,3-triazoles exhibit very good selective inhibition toward butyrylcholinesterase (BChE), while the same derivatives converted into salts also display inhibitory activity against acetylcholinesterase (AChE), enzymes relevant to Alzheimer’s disease therapy. They show even better BChE inhibition potential than neutral analogs. Methods: This study presents the synthesis and biological evaluation of a novel series of charged thienobenzo-1,2,3-triazolinium salts (**1**–**17**) as inhibitors of AChE and BChE. The basic skeleton of the targeted compounds was synthesized via a photochemical method and subsequently converted into corresponding bromide salts. Their structures were confirmed using NMR and HRMS analyses. Results: In vitro testing showed that all synthesized compounds exhibit moderate to strong BChE inhibition and, to a lesser extent, AChE inhibition. Compounds **8** and **11** emerged as the most potent AChE inhibitors (IC_50_ ~ 2.6–3.2 µM), while compounds **1**, **2**, and **8** demonstrated excellent and selective BChE inhibition (IC_50_ ~ 0.3–0.4 µM), outperforming the reference drug galantamine. Anti-inflammatory evaluation revealed limited activity, with compound **17** slightly reducing LPS-induced TNF-α production at the highest tested concentration. Conclusions: These findings highlight the role of the electric charge and substituent type in modulating biological activity and confirm the therapeutic potential of these molecules as dual cholinesterase inhibitors for further development in neurodegenerative disease treatment.

## 1. Introduction

**Figure 1:**
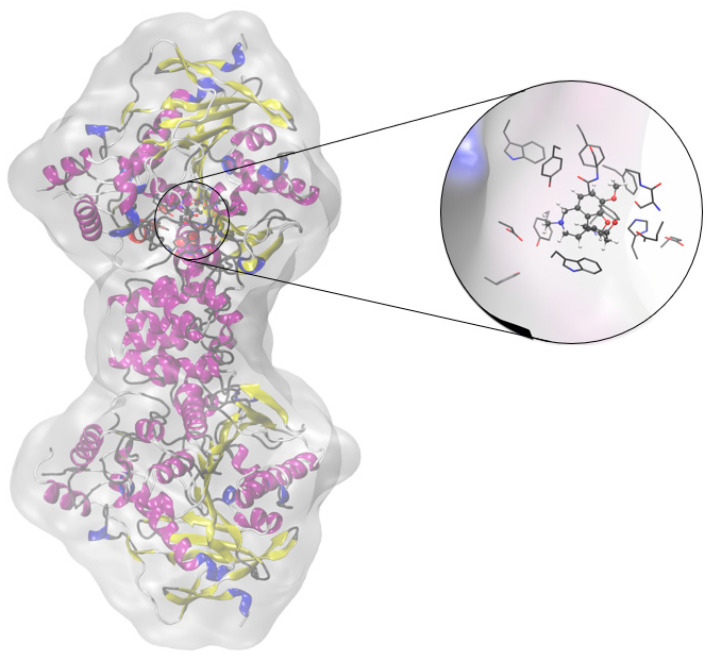
Crystal structure of the human AChE enzyme in complex with galantamine (crystal structure 4EY6.pdb [5]).

Cholinesterase enzymes are divided into two groups: acetylcholinesterase (AChE; EC 3.1.1.7) and butyrylcholinesterase (BChE; EC 3.1.1.8) [1]. AChE is primarily located at postsynaptic neuromuscular junctions, i.e., in muscles and nerves, and is also associated with erythrocytes in the blood [2,3]. On the other hand, BChE is synthesized in the liver and is found in blood plasma and various organs [4]. Both enzymes consist of 12 *β*-sheets surrounded by 14 *α*-helices. The active site gorge of cholinesterases is 20 Å deep and 5 Å wide. The volume of the active site cavity in BChE is 500 Å^3^, whereas in AChE, it is 300 Å^3^ (Figure 1). BChE has a larger volume because AChE contains 14 aromatic amino acids within its active site, 6 of which are replaced by aliphatic amino acids in BChE. This structural difference makes BChE a less specific enzyme. The variation in amino acids within the active site prevents some molecules from inhibiting both enzymes simultaneously [2,4].

AChE primarily functions to hydrolyze acetylcholine (ACh) into acetic acid and choline. Some of its additional roles include the modulation of cerebral blood flow, *β*-amyloid aggregation, activation and expression of the APP95 protein, τ protein phosphorylation, and the regulation of inflammatory processes. On the other hand, BChE can hydrolyze acetylcholine as well as other choline esters. However, the rate of acetylcholine hydrolysis by BChE is slower than that by AChE. The exact physiological role of BChE remains unclear, but its pharmacological and toxicological importance has been recognized [1,2,4]. Both enzymes play a key role in the cholinergic anti-inflammatory pathway, which links nerve endings with macrophages through the α7 nicotinic acetylcholine receptor (α7 nAChR) on their surface. The discovery of this pathway led to the understanding that the central nervous system regulates innate immunity. Erythrocyte-bound AChE acts as a switch for activating this pathway. The cholinergic anti-inflammatory pathway operates through the following mechanism: the vagus nerve releases ACh, which activates α7 nAChR, resulting in the opening of the central channel and the influx of Ca^2+^ ions into macrophages. The increased intracellular Ca^2+^ concentration activates the NF-κB factor, which subsequently inhibits the secretion of pro-inflammatory cytokines such as TNF-α and IL-6. AChE and BChE serve as regulators of this pathway by degrading acetylcholine [1].

High levels of BChE have been found to be associated with brain plaques and neurofibrillary tangles, which are neuropathological features of Alzheimer’s disease (AD) [6,7,8]. Therefore, both cholinesterases are pharmacologically relevant targets in neurodegenerative diseases, and current treatment includes cholinesterase inhibitors such as donepezil, galantamine, physostigmine, rivastigmine, etc. [9,10,11,12,13,14,15,16]. However, none of the approved drugs act on the pathophysiological factors of AD; they only alleviate its symptoms. Thus, many other molecules that act as cholinesterase enzyme inhibitors can also be considered potential therapeutic agents for AD [17,18,19,20,21,22].

**Figure 2:**
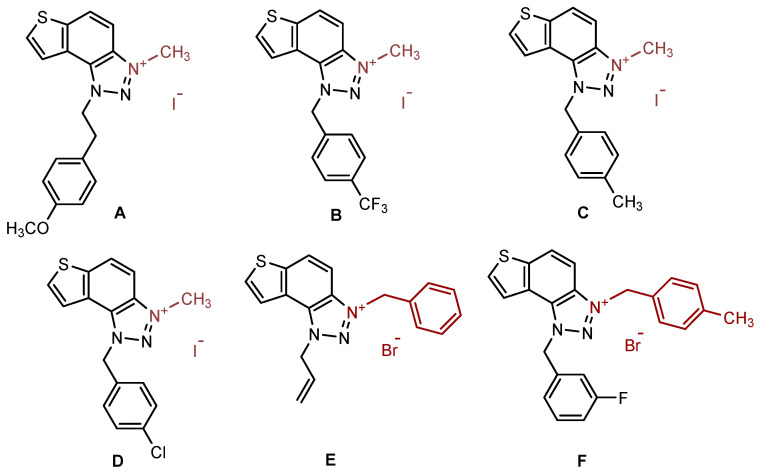
Structures of proven dual cholinesterase inhibitors A–F based on the charged thienobenzo-1,2,3-triazole skeleton.

Based on previously synthesized compounds, it has been shown that thienobenzo-1,2,3-triazoles exhibit very good selective inhibition toward BChE, while the same derivatives converted into salts also display inhibitory activity against AChE, showing even better BChE inhibition potential than neutral analogs [23,24,25,26,27]. This finding supported the importance of the electric charge in biological processes and compound activity [28]. The first reported triazolinium salts were synthesized using methyl iodide [27,29]. The most potent inhibitory effects were observed for compounds **A**–**D** (Figure 2) with IC_50_ values better than the standard galantamine for BChE and somewhat weaker for AChE. The most important structural feature for thienobenzo-triazoles is the presence of the charge. In recent research [30], the most potent compound was thienobenzo-1,2,3-triazolinium salt **E**, which inhibited BChE with an IC_50_ of 98 nM, while bromide salt **F** also displayed significant anti-inflammatory activity by inhibiting LPS-induced TNF-α production (IC_50_ = 0.66 μM). Those promising in vitro and in silico results of thienobenzo-1,2,3-triazole-based salts **A**–**F** (Figure 2) highlighted the importance of further structural charge modulation in optimizing cholinesterase inhibitors that will offer even better dual therapeutic functions. Motivated by this, the present study introduces a new series of triazolinium bromide salts.

## 2. Results and Discussion

### 2.1. Photochemically Assisted Synthesis of Charged Thienobenzo-1,2,3-Triazoles ***1***–***17***

**Scheme 1:**
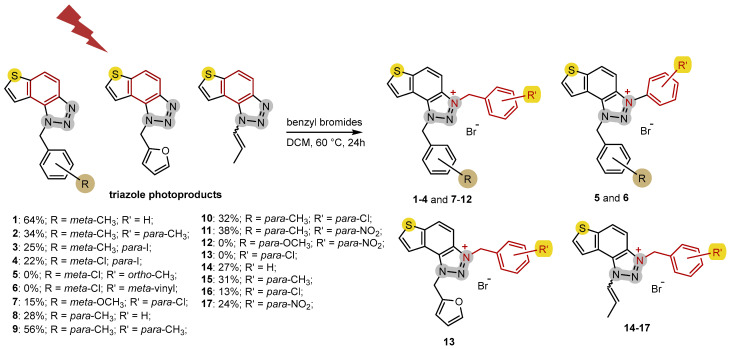
Final synthetic step to charged triazolinium benzyl salts **1**–**17** from triazole photoproducts and their isolated yields are listed.

To obtain the charged thienobenzo-1,2,3-triazoles **1**–**17**, a series of four consecutive reactions were carried out. The 1,4-disubstituted triazole aldehydes as starting reagents in this research were prepared according to the known procedure [31]. Aldehydes gave the corresponding triazolo-stilbenes in the Wittig reaction with the phosphonium salt. Thienobenzo-triazoles as starting compounds for the preparation of the targeted bromide salts **1**–**17** (Scheme 1) were synthesized by photochemical cyclization reactions from triazolo-stilbenes using a wavelength of 300 nm. Each triazole photoproduct (Scheme 1) was dissolved in dichloromethane and briefly purged with argon to maintain an inert atmosphere. Subsequently, 20 equivalents of the corresponding benzyl bromide were added. At the end, to the cooled reaction, diethyl ether was added to induce precipitation. The resulting mixture was centrifuged, the supernatant was decanted, and the remaining solid bromide salts **1**–**4**, **7**–**11,** and **14**–**17** were dried using a rotary evaporator. Salts **5**, **6**, **12,** and **13** did not precipitate from the reaction mixture, probably due to the nature of the starting bromide, the instability of the individual derivative, and/or the nature and position of the substituents.

**Figure 3:**
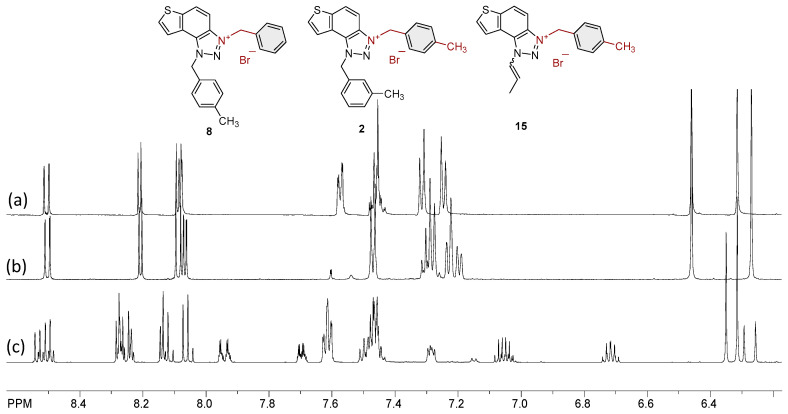
Crucial parts of the ^1^H NMR (CD_3_OD) spectra of the thienobenzo-1,2,3-triazolinium salts: **8** (**a**), **2** (**b**), and **15** (**c**).

All the synthesized thienobenzo-1,2,3-triazolinium salts **1**–**17** have been fully proven by NMR and HRMS analyses (Figure 3). In the ^1^H NMR spectra of triazolinium salts **1**–**17**, a new signal of the second methylene group on the triazole nitrogen is visible between 6.1 and 6.4 ppm, undoubtedly confirming the formation of the targeted charged structures **8**, **2**, and **15** (Figure 3), beside the other signals.

### 2.2. Cholinesterase Inhibition Activity of Triazolinium Salts ***1***–***17***

Building on the previously promising in vitro results of thienobenzo-1,2,3-triazolinium salts against cholinesterases [29,30], research was extended to a series of structurally related bromide salts. The inhibitory effects of a new set of triazolinium salts (**1**–**4**, **7**–**11**, **14**–**17**) on the activities of acetylcholinesterase (AChE) and butyrylcholinesterase (BChE) are summarized in Table 1. Their inhibitory activity was assessed using a modified Ellman method [32] over a wide concentration range (0.01–250 μM). The IC_50_ values listed in Table 1 are relative IC_50_ values, obtained directly from the nonlinear regression analysis of the inhibition curves under defined test conditions. For reference, the inhibitory effect at the highest tested concentration is also reported. The results were compared with galantamine, a well-known cholinesterase inhibitor used in the treatment of Alzheimer’s disease.

All tested trazolinium salts showed inhibitory effects on AChE in the micromolar (μM) range concentrations. Compounds **8** (IC_50_ = 2.6 μM) and **11** (IC_50_ = 3.2 μM) stand out as the most potent AChE inhibitors. These compounds share the same substituent (*p*-methylbenzyl) on the charged thienobenzo-1,2,3-triazole ring. After them, a slightly weaker inhibitory effect was achieved by the following compounds: **1**, **2**, **3, 9**, **10**, **14**, and **16**, with IC_50_ values for AChE from 4.1 to 6.5 μM. Their IC_50_ values are significantly lower compared to the remaining compounds (**4**, **7**, **15**, and **17**). It is important to note that all the tested compounds are less potent than galantamine in inhibiting AChE (IC_50_ = 0.15 μM). All of the triazolinium salts that were evaluated can be classified as moderate AChE inhibitors, with the exception of compound **17**, which is a weak AChE inhibitor. The structure of compound **17** with nitrobenzyl and propenyl groups on the triazole ring seems to reduce its ability to interact with AChE. Meanwhile, compounds **14**, **15**, and **16** with benzyl, methylbenzyl, and chlorobenzyl substituents show significantly stronger inhibitory effects.

It is interesting that all the tested triazolinium salts, except one, showed better BChE inhibition than the reference galantamine, with IC_50_ values from 0.3 to 3.5 μM (Table 1). Among the tested compounds, derivatives **1**, **2**, and **8**, which have benzyl and methylbenzyl groups on the triazole ring, and compounds **3** and **4** with benzyl substituents (with –I, –Cl, or –CH_3_) stand out as the most potent and selective BChE inhibitors. A similar finding was confirmed in previous research [30]. Compound **17** also inhibits BChE, but it is significantly less potent compared to the other tested compounds, similar to its activity on AChE.

In conclusion, although the newly synthesized charged thienobenzo-1,2,3-triazolinium salts (**1**–**17**) can be considered as dual cholinesterase inhibitors, they all showed stronger inhibitory activity on BChE. The percentage of inhibition for most compounds is around 75–85% at certain concentrations, which indicates a significant decrease in enzyme activity. The strongest dual inhibitor is compound **8**. The dose–response curves for AChE (a) and BChE (b) inhibition for compounds **8** and **1** are shown in Figure 4 and Figure 5 and of all other investigated compounds on Figures S1–S13.

The preferential inhibition of BChE by certain compounds makes them promising candidates for further investigation in the context of neurodegenerative diseases—particularly Alzheimer’s disease—where selective AChE inhibitors are sought for early stages and dual inhibitors for later stages.

### 2.3. Anti-Inflammatory Activity of Triazolinium Salts ***1***–***17***

**Figure 6:**
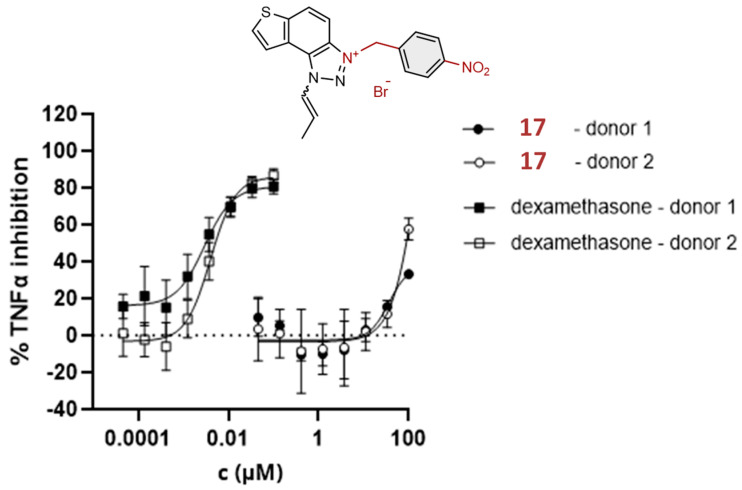
Inhibition of LPS-stimulated TNFα production in PBMCs from two donors for charged triazolinium bromide salt **17**.

The potential anti-inflammatory activity of compounds **1**–**4**, **7**–**11**, and **14**–**17** was also evaluated in vitro by the measurement of TNFα production in LPS-stimulated PBMCs. Most of the tested compounds reduced cell viability at the highest tested concentrations. At lower concentrations that were not affecting cell viability, LPS-induced TNFα production was not changed. However, thienobenzo-1,2,3-triazolinium salt **17** had no effect on cell viability and it slightly inhibited LPS-stimulated TNFα production (Figure 6). Compound **17** was active only at the highest tested concentration.

A corticosteroid widely used for the treatment of inflammatory conditions (dexamethasone) was used as a reference compound in this assay. It inhibited LPS-stimulated TNFα production with an IC_50_ value of 3.5 nM in PBMCs from two donors. High potency of dexamethasone was expected and was in line with results previously obtained in this assay [25]. From this result, it is not possible to link the inhibitory activity of the salts **1**–**17** toward cholinesterases and their anti-inflammatory effect.

## 3. Materials and Methods

### 3.1. General Remarks

NMR spectra were recorded using either a Bruker AV300 or AV600 spectrometer (Bruker BioSpin GmbH, Rheinstetten, Germany) equipped with a 5 mm probe. Standard ^1^H and proton-decoupled ^13^C{^1^H} NMR spectra were collected at operating frequencies of 600.130 MHz for ^1^H and 75.432 or 150.903 MHz for ^13^C. Chemical shifts (*δ*, in ppm) were referenced to the signal from tetramethylsilane (TMS). All measurements were performed in deuterated chloroform (CD_3_OD) at 25 °C. Photochemical reactions were conducted in 50.0 mL solutions contained in quartz cuvettes that permitted light transmission. A Luzchem photoreactor equipped with 16 UV lamps (emitting at 300 nm) was used for irradiation. All solvents used were commercially sourced and purified by distillation. Phosphonium salts were synthesized in-house, and the compound 1-(4-nitrophenyl)-1*H*-1,2,3-triazole-4-carbaldehyde had also been previously prepared in our lab [31]. Reaction progress was monitored by thin-layer chromatography (TLC) using 0.2 mm silica gel-coated plates (60/Kieselguhr F254) and an appropriate solvent system in 10 mL volumes. After each reaction, the mixture was cooled to 0 °C and treated with diethyl ether to induce product precipitation. The suspension was centrifuged using a Centrifuge Eba 20 (Hettich, Tuttlingen, Germany), firstly at 2 × 3000 rpm for 10 min, then at 5 × 5000 rpm for another 10 min. The supernatant was decanted, and the remaining material was evaporated. High-resolution mass spectrometry (HRMS) was performed on a MALDI TOF/TOF instrument using an Nd:YAG laser (355 nm) operating at a repetition rate of 200 Hz.

### 3.2. Synthesis of Bromide Salts ***1***–***17***

Triazole-based photoproducts (Scheme 1), previously developed by our research group [23,24,25,26], were used as precursors for the synthesis of triazolinium salts **1**–**17**. Each triazole derivative was dissolved in 0.6 mL of dry dichloromethane (DCM) in a small reaction vial and briefly purged with argon to maintain an inert atmosphere. Subsequently, 20 equivalents of the corresponding benzyl bromide were added. The reaction mixture was stirred in an oil bath at 60 °C for 24 h. After completion, the reaction was cooled to 0 °C, and approximately 5 mL of diethyl ether was added to induce precipitation, mostly forming a white suspension. The resulting mixture was centrifuged at 2 × 3000 rpm for 10 min, followed by 5 × 5000 rpm for an additional 10 min. The supernatant was decanted, and the remaining solid was dried using a rotary evaporator. NMR analysis confirmed the successful synthesis of bromide salts **1**–**17**.


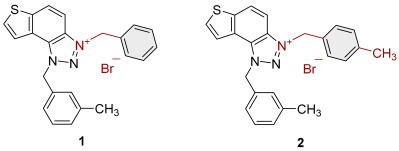


3-benzyl-1-(3-methylbenzyl)-1*H*-thieno[3′,2′:3.4]benzo[1.2-*d*][1.2,3]triazol-3-ium bromide(**1**): 6.2 mg (64% isolated), white powder; m.p. 113–114 °C; ^1^H NMR (CD_3_OD, 600 MHz) *δ*/ppm: 8.51 (d, *J* = 9.2 Hz, 1H), 8.21 (d, *J* = 5.4 Hz, 1H), 8.10 (d, *J* = 9.2 Hz, 1H), 8.07 (dd, *J* = 5.6 Hz, 1H), 7.49–7.41(m, 7H), 7.30 (t, *J* = 7.8 Hz, 1H), 7.20 (d, *J* = 7.8 Hz, 1H), 6.47 (s, 2H), 6.32 (s, 2H), 2.32 (s, 3H); ^13^C NMR (CD_3_OD, 75 MHz) *δ*/ppm: 144.4, 141.9, 140.8, 134.4, 133.6, 133.3, 131.2, 130.8, 130.53, 130.49, 130.46, 130.43, 129.9, 129.6, 129.3, 128.4, 125.9, 124.6, 122.0, 109.2, 57.5, 56.7, 21.3; HRMS (ESI) (*m*/*z*) za C_23_H_20_N_3_S^+^ Br^−^: [M + H]^+^_calcd_ = 370.1378, and [M + H]^+^_measured_ = 370.1376.

1-(3-methylbenzyl)-3-(4-methylbenzyl)-1*H*-thieno[3′,2′:3.4]benzo[1.2-*d*][1.2,3]triazol-3-ium bromide (**2**): 3.4 mg (34% isolated), white powder; m.p. 116–117 °C; ^1^H NMR (CD_3_OD, 600 MHz) *δ*/ppm: 8.50 (dd, *J* = 9.3 Hz, 1H), 8.21 (d, *J* = 5.5 Hz, 1H), 8.09 (d, *J* = 9.3 Hz, 1H), 8.07 (d, *J* = 5.5 Hz, 1H), 7.47 (d, *J* = 8.2 Hz, 2H), 7.31–7.27 (m, 3H), 7.24–7.18 (m, 3H), 6.46 (s, 2H), 6.27 (s, 2H), 2.36 (s, 3H), 2.32 (s, 3H). ^13^C NMR (CD_3_OD, 75 MHz) *δ*/ppm: 144.3, 141.1, 140.7, 138.9, 138.7, 135.4, 134.4, 133.4, 132.3, 131.2, 131.1, 130.4, 130.0, 129.9, 129.3, 128.3, 125.8, 124.6, 122.0, 109.3, 57.5, 56.5, 21.4, 21.2; HRMS (ESI) (*m*/*z*) za C_24_H_22_N_3_S^+^ Br^−^: [M + H]^+^_calcd_ = 384.1534, and [M + H]^+^_measured_ = 384.1531.


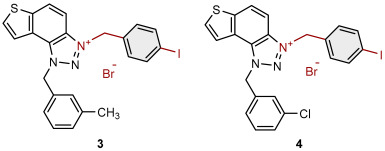


3-(4-iodobenzyl)-1-(3-methylbenzyl)-1*H*-thieno[3′,2′:3.4]benzo[1.2-*d*][1.2,3]triazol-3-ium bromide (**3**): 1.8 mg (25% isolated), white powder; m.p. 122–124 °C; ^1^H NMR (CD_3_OD, 600 MHz) *δ*/ppm: 8.53 (dd, *J* = 9.2 Hz, 1H), 8.22 (d, *J* = 5.6 Hz, 1H), 8.11 (d, *J* = 9.2 Hz, 1H), 8.08 (d, *J* = 5.6 Hz, 1H), 7.58 (dt, *J* = 8.5, 1.8 Hz, 2H), 7.48 (dt, *J* = 8.5, 1.8 Hz, 2H), 7.31 (t, *J* = 7.8 Hz, 1H), 7.24 (s, 1H), 7.23 (s, 1H), 7.20 (d, *J* = 7.8 Hz, 1H), 6.46 (s, 2H), 6.31 (s, 2H), 2.32 (s, 3H); ^13^C NMR (CD_3_OD, 75 MHz) *δ*/ppm: 144.5, 138.9, 135.6, 134.5, 133.2, 132.3, 131.7, 131.2, 130.6, 130.4, 129.4, 128.5, 126.0, 124.6, 122.0, 109.1, 57.6, 55.8, 21.2 (signals for 4 quaternary C are missing); HRMS (ESI) (*m*/*z*) za C_23_H_19_IN_3_S^+^ Br^−^: [M + H]^+^_calcd_ = 497.0311, and [M + H]^+^_measured_ = 497.0317.

1-(3-chlorobenzyl)-3-(4-iodobenzyl)-1*H*-thieno[3′,2′:3.4]benzo[1.2-*d*][1.2,3]triazol-3-ium bromide (**4**): 2.2 mg (22% isolated), yellowish powder; m.p. 117–118 °C; ^1^H NMR (CD_3_OD, 600 MHz) *δ*/ppm: 8.55 (d, *J* = 9.3 Hz, 1H), 8.26 (d, *J* = 8.3 Hz, 1H), 8.13–8.10 (m, 3H), 7.84 (t, *J* = 8.5, 1H), 7.82 (t, *J* = 8.5, 1H), 7.52 (t, *J* = 1.6 Hz, 1H), 7.43 (t, *J* = 8.0 Hz, 1H), 7.39–7.36 (m, 1H), 7.35 (t, *J* = 1.7 Hz, 1H), 7.34 (t, *J* = 1.7 Hz, 1H), 6.51 (s, 2H), 6.27 (s, 2H). ^13^C NMR (CD_3_OD, 75 MHz) *δ*/ppm: 144.6, 139.8, 136.8, 136.4, 135.6, 135.4, 135.3, 134.7, 132.4, 132.1, 131.9, 130.7, 130.6, 129.3, 129.2, 128.6, 127.6, 124.5, 121.8, 109.1 (signals for 2 quaternary C are missing); HRMS (ESI) (*m*/*z*) za C_22_H_16_ClIN_3_S^+^ Br^−^: [M + H]^+^_calcd_ = 515.9798, and [M + H]^+^_measured_ = 515.9787.


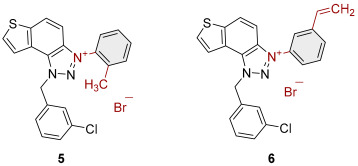


1-(3-chlorobenzyl)-3-(*o*-tolyl)-1*H*-thieno[3′,2′:3.4]benzo[1.2-*d*][1.2,3]triazol-3-ium bromide (**5**): with the addition of ether, no precipitate was formed. ^1^H NMR confirms that no product was formed, only the initial uncharged photoproduct is visible.

1-(3-chlorobenzyl)-3-(3-vinylphenyl)-1*H*-thieno[3′,2′:3.4]benzo[1.2-*d*][1.2,3]triazol-3-ium bromide (**6**): with the addition of ether, no precipitate was formed. ^1^H NMR confirms that no product was formed, only the initial uncharged photoproduct is visible.


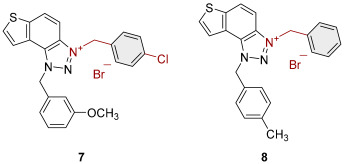


3-(4-chlorobenzyl)-1-(3-methoxybenzyl)-1*H*-thieno[3′,2′:3.4]benzo[1.2-*d*][1.2,3]triazol-3-ium bromide (**7**): 1.3 mg (15% isolated), orange powder; m.p. 125–126 °C; ^1^H NMR (CD_3_OD, 600 MHz) *δ*/ppm: 8.54 (dd, *J* = 9.2 Hz, 1H), 8.23 (d, *J* = 5.6 Hz, 1H), 8.12 (d, *J* = 9.2 Hz, 1H), 8.08 (d, *J* = 5.6 Hz, 1H), 7.58 (dt, *J* = 8.4 Hz, 2H), 7.48 (dt, *J* = 8.7 Hz, 2H), 7.35–7.31 (m, 1H), 6.99–6.96 (m, 2H), 6.94 (d, *J* = 7.7 Hz, 1H), 6.47 (s, 2H), 6.32 (s, 2H), 3.76 (s, 3H). ^13^C NMR (CD_3_OD, 150 MHz) *δ*/ppm: 160.5, 143.1, 135.4, 134.1, 133.3, 133.1, 130.8, 130.3, 129.2, 127.1, 123.2, 120.6, 119.3, 114.3, 113.3, 107.7, 56.0, 54.4, 54.3 (signals for 4 quaternary C are missing); HRMS (ESI) (*m*/*z*) za C_23_H_19_ClN_3_OS^+^ Br^−^: [M + H]^+^_calcd_ = 421.0121, and [M + H]^+^_measured_ = 421.0117.

3-benzyl-1-(4-methylbenzyl)-1*H*-thieno[3′,2′:3.4]benzo[1.2-*d*][1.2,3]triazol-3-ium bromide (**8**): 11.7 mg (28% isolated) white powder; m.p. 107–108 °C; ^1^H NMR (CD_3_OD, 600 MHz) *δ*/ppm: 8.51 (d, *J* = 9.2 Hz, 1H), 8.21 (d, *J* = 5.6 Hz, 1H), 8.09 (d, *J* = 9.2 Hz, 1H), 8.08 (dd, *J* = 5.6 Hz, 1H), 7.57 (dd, *J* = 8.0 Hz, 2H), 7.48–7.44 (m, 3H), 7.32 (d, *J* = 8.1 Hz, 2H), 7.25 (d, *J* = 8.1 Hz, 2H), 6.46 (s, 2H), 6.32 (s, 2H), 2.34 (s, 3H). ^13^C NMR (CD_3_OD, 150 MHz) *δ*/ppm: 143.0, 139.4, 134.1, 133.0, 132.1, 130.9, 129.7, 129.4, 129.1, 128.9, 128.5, 127.5, 127.0, 123.2, 120.6, 107.8, 56.0, 55.3, 19.7 (signals for 4 quaternary C are missing); HRMS (ESI) (*m*/*z*) za C_23_H_20_N_3_S^+^ Br^−^: [M + H]^+^_calcd_ = 370.1378, and [M + H]^+^_measured_ = 370.1373.


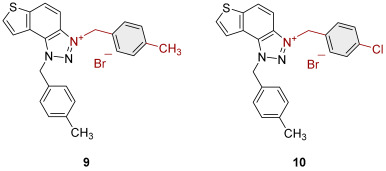


1.3-bis(4-methylbenzyl)-1*H*-thieno[3′,2′:3.4]benzo[1.2-*d*][1.2,3]triazol-3-ium bromide (**9**): 4.2 mg (56% isolated), white powder; m.p. 105–107 °C; ^1^H NMR (CD_3_OD, 600 MHz) *δ*/ppm: 8.49 (d, *J* = 9.2 Hz, 1H), 8.20 (d, *J* = 5.6 Hz, 1H), 8.09–8.06 (m, 2H), 7.46 (d, *J* = 8.1 Hz, 2H), 7.31 (d, *J* = 8.1 Hz, 2H), 7.28 (d, *J* = 8.1 Hz, 2H), 7.25 (d, *J* = 8.1 Hz, 2H), 6.45 (s, 2H), 6.26 (s, 2H), 2.36 (s, 3H), 2.34 (s, 3H). ^13^C NMR (CD_3_OD, 75 MHz) *δ*/ppm: 144.4, 141.1, 140.8, 135.4, 134.3, 132.3, 131.11, 131.09, 130.5, 130.4, 129.9, 128.9, 128.3, 124.6, 122.0, 109.3, 57.4, 56.6, 21.2, 21.1 (signals for 4 quaternary C are missing); HRMS (ESI) (*m*/*z*) za C_24_H_22_N_3_S^+^ Br^−^: [M + H]^+^_calcd_ = 384.1534, and [M + H]^+^_measured_ = 384.1531.

3-(4-chlorobenzyl)-1-(4-methylbenzyl)-1*H*-thieno[3′,2′:3.4]benzo[1.2-*d*][1.2,3]triazol-3-ium bromide (**10**): 2.8 mg (32% isolated), orange powder; m.p. 100–101 °C; ^1^H NMR (CD_3_OD, 600 MHz) *δ*/ppm: 8.53 (d, *J* = 9.2 Hz, 1H), 8.22 (d, *J* = 5.6 Hz, 1H), 8.10 (d, *J* = 9.2 Hz, 1H), 8.09 (dd, *J* = 5.4 Hz, 1H), 7.57 (dt, *J* = 8.6 Hz, 2H), 7.48 (dt, *J* = 8.6 Hz, 2H), 7.32 (d, *J* = 8.1 Hz, 2H), 7.25 (d, *J* = 8.1 Hz, 2H), 6.45 (s, 2H), 6.30 (s, 2H), 2.34 (s, 3H). ^13^C NMR (CD_3_OD, 75 MHz) *δ*/ppm: 144.4, 140.9, 136.8, 135.5, 134.4, 132.3, 132.2, 131.7, 131.1, 130.6, 130.3, 128.9, 128.5, 124.7, 122.0, 109.1, 57.5, 55.8, 21.1 (signals for 4 quaternary C are missing); HRMS (ESI) (*m*/*z*) za C_23_H_19_ClN_3_S^+^ Br^−^: [M + H]^+^_calcd_ = 404.0980, and [M + H]^+^_measured_ = 404.0988.


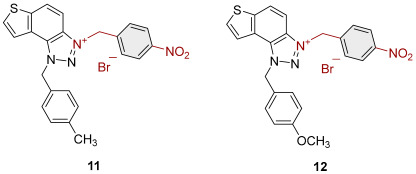


1-(4-methylbenzyl)-3-(4-nitrobenzyl)-1*H*-thieno[3′,2′:3.4]benzo[1.2-*d*][1.2,3]triazol-3-ium bromide (**11**): 3.0 mg (38% isolated), white powder; m.p. 120–121 °C; ^1^H NMR (CD_3_OD, 600 MHz) *δ*/ppm: 8.55 (d, *J* = 9.2 Hz, 1H), 8.32 (d, *J* = 8.6 Hz, 2H), 8.24 (d, *J* = 5.7 Hz, 1H), 8.13–8.10 (m, 2H), 7.79 (d, *J* = 8.6 Hz, 2H), 7.34 (d, *J* = 8.1 Hz, 2H), 7.25 (d, *J* = 8.1 Hz, 2H), 6.47 (s, 4H), 2.34 (s, 3H). ^13^C NMR (CD_3_OD, 150 MHz) *δ*/ppm: 148.6, 148.3, 143.2, 143.1, 139.5, 138.9, 134.4, 133.2, 131.0, 129.9, 129.7, 129.6, 128.7, 127.6, 127.3, 123.9, 123.4, 123.3, 120.6, 107.6, 56.2, 54.0, 19.7; HRMS (ESI) (*m*/*z*) za C_23_H_19_N_4_O_2_S^+^ Br^−^: [M + H]^+^_calcd_ = 415.1229, and [M + H]^+^_measured_ = 415.1223.

1-(4-methoxybenzyl)-3-(4-nitrobenzyl)-1*H*-thieno[3′,2′:3.4]benzo[1.2-*d*][1.2,3]triazol-3-ium bromide (**12**): with the addition of ether, no precipitate was formed. ^1^H NMR confirms that no product was formed, only the initial uncharged photoproduct is visible.


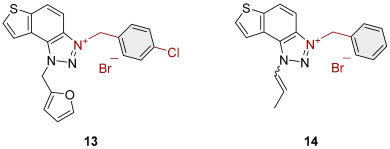


3-(4-chlorobenzyl)-1-(furan-2-ylmethyl)-1*H*-thieno[3′,2′:3.4]benzo[1.2-*d*][1.2,3]triazol-3-ium bromide (**13**): with the addition of ether, no precipitate was formed. ^1^H NMR confirms that no product was formed, only the initial uncharged photoproduct is visible.

3-benzyl-1-(prop-1-en-1-yl)-1*H*-thieno[3′,2′:3.4]benzo[1.2-*d*][1.2,3]triazol-3-ium bromide (**14**): 2.0 mg (27% isolated), white powder; mixture of *cis*- and *trans*-isomer (^1^H NMR, *cis*-:*trans*- = 1:7):

*cis*-**14:** ^1^H NMR (CD_3_OD, 600 MHz) *δ*/ppm: 8.54 (dd, *J* = 9.2 Hz, 1H), 8.27 (d, *J* = 5.6 Hz, 1H), 8.14 (d, *J* = 5.6 Hz, 1H), 8.12 (d, *J* = 9.2 Hz, 1H), 7.69 (dq, *J* = 8.4, 1.8 Hz, 1H), 7.63–7.59 (m, 2H), 7.49–7.43 (m, 3H), 6.71 (dq, *J* = 8.7 Hz, 1H), 6.34 (s, 2H), 1.91 (dd, *J* = 7, 1.8 Hz, 3H);

*trans*-**14:** ^1^H NMR (CD_3_OD, 600 MHz) *δ*/ppm: 8.50 (dd, *J* = 9.2 Hz, 1H), 8.28 (d, *J* = 5.6 Hz, 1H), 8.24 (d, *J* = 5.6 Hz, 1H), 8.06 (d, *J* = 9.2 Hz, 1H), 7.94 (dq, *J* = 13.5, 1.8 Hz, 1H), 7.63–7.59 (m, 2H), 7.49–7.43 (m, 3H), 7.05 (dq, *J* = 13.5, 7 Hz, 1H), 6.31 (s, 2H), 2.20 (dd, *J* = 7, 1.8 Hz, 3H);

HRMS (ESI) (*m*/*z*) za C_18_H_16_N_3_S^+^ Br^−^: [M + H]^+^_calcd_ = 306.1065, and [M + H]^+^_measured_ = 306.1061.


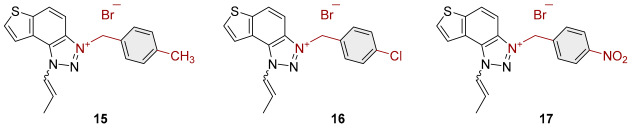


3-(4-methylbenzyl)-1-(prop-1-en-1-yl)-1*H*-thieno[3′,2′:3.4]benzo[1.2-*d*][1.2,3]triazol-3-ium (**15**): 11.7 mg (31% isolated), white powder; mixture of *cis*- and *trans*-isomer (^1^H NMR, *cis*-:*trans*- = 1:1.2). In the ^1^H NMR spectrum, key signals are visible in the expected regions, but due to significant overlap between *cis-* and *trans-*isomers, it is difficult to assign individual signals. HRMS (ESI) (*m*/*z*) za C_19_H_18_N_3_S^+^ Br^−^: [M + H]^+^_calcd_ = 320.1221, and [M + H]^+^_measured_ = 320.1221.

3-(4-chlorobenzyl)-1-(prop-1-en-1-yl)-1*H*-thieno[3′,2′:3,4]benzo[1,2-*d*][1–3]triazol-3-ium (**16**): 5.2 mg (13% isolated), white powder; mixture of *cis*- and *trans*-isomer (^1^H NMR, *cis*-:*trans*- = 1:1.25):

*cis*-**16** ^1^H NMR (CD_3_OD, 600 MHz) *δ*/ppm: 8.56–8.49 (m, 1H), 8.30–8.23 (m, 1H), 8.15–8.05 (m, 2H), 7.70 (dq, *J* = 8.4, 1.8 Hz, 1H), 7.63–7.59 (m, 2H), 7.50–7.40 (m, 2H), 6.74–6.69 (dq, *J* = 8.4, 7.2 Hz, 1H), 6.35 (s, 2H), 1.91 (dd, *J* = 7.2, 1.8 Hz, 3H);

*trans*-**16** ^1^H NMR (CD_3_OD, 600 MHz) *δ*/ppm: 8.56–8.49 (m, 1H), 8.30–8.23 (m, 2H), 8.15–8.05 (m, 1H), 7.94 (dq, *J* = 13.5, 1.8 Hz, 1H), 7.63–7.59 (m, 2H), 7.50–7.40 (m, 2H), 7.09–7.02 (dq, *J* = 13.5, 7.2 Hz, 1H), 6.31 (s, 2H), 2.20 (dd, *J* = 7, 1.8 Hz, 3H);

HRMS (ESI) (*m*/*z*) za C_18_H_15_ClN_3_S^+^ Br^−^: [M + H]^+^_calcd_ = 340.0675, and [M + H]^+^_measured_ = 340.0669.

3-(4-nitrobenzyl)-1-(prop-1-en-1-yl)-1*H*-thieno[3′,2′:3,4]benzo[1,2-*d*][1–3]triazol-3-ium (**17**): 2.0 mg (24% isolated), white powder; mixture of *cis*- and *trans*-isomer (^1^H NMR, *cis*-:*trans*- = 1:10):

*cis*-**17** ^1^H NMR (CD_3_OD, MHz) *δ*/ppm: 8.58 (d, *J* = 9.2 Hz, 1H), 8.35–8.29 (m, 3H), 8.17 (d, *J* = 5.6 Hz, 1H), 8.16–8.14 (m, 1H), 7.84–7.80 (m, 2H), 6.51 (s, 2H), 1.92 (dd, *J* = 7.2, 1.8 Hz, 3H), other signals are not visible (because of small amount of *cis*-**17** isomer in the mixture);

*trans*-**17** ^1^H NMR (CD_3_OD, MHz) *δ*/ppm: 8.55 (d, *J* = 9.2 Hz, 1H), 8.35–8.29 (m, 3H), 8.27 (d, *J* = 5.6 Hz, 1H), 8.10–8.07 (m, 1H), 7.98–7.95 (dq, *J* = 13.5, 1.8 Hz, 1H), 7.84–7.80 (m, 2H), 7.09–7.03 (dq, *J* = 13.5, 7.2 Hz, 1H), 6.47 (s, 2H), 2.20 (dd, *J* = 7.2, 1.8 Hz, 3H);

HRMS (ESI) (*m*/*z*) za C_18_H_15_N_4_O_2_S^+^ Br^−^: [M + H]^+^_calcd_ = 352.0902, and [M + H]^+^_measured_ = 352.0908.

### 3.3. In Vitro Cholinesterase Inhibition Activity Measurements of Bromide Salts ***1***–***17***

The inhibitory effects of the new synthesized thiabenzo-1,2,3-triazolinium bromide salts, **1**–**4**, **7**–**11,** and **14**–**17**, on acetylcholinesterase (AChE) and butyrylcholinesterase (BChE) activity were evaluated using the modified Ellman’s method [32]. Ellman’s reagent (DTNB, 5,5’-dithiobis-(2-nitrobenzoic acid)), AChE (derived from electric eel, type VI-S), BChE (extracted from equine serum), acetylthiocholine iodide (ATChI), S-butyrylthiocholine iodide (BTChI), galantamine hydrobromide, Tris-HCl buffer, and 96% ethanol were acquired from Sigma-Aldrich (St. Louis, MO, USA).

DTNB, ATChI, and BTChI were prepared in 50 mM Tris buffer (pH 8.0), while the enzymes were made in 20 mM Tris buffer (pH 7.5). Using a 96-well microplate reader (Agilent, BioTek 800TS, Santa Clara, CA, USA), cholinesterase activity was assessed. Amounts of 180 μL of 50 mM Tris buffer, 10 μL of tested solutions with final concentrations ranging from 0.01 to 250 μM, and 10 μL of an enzyme with a final concentration of 0.03 U/mL were added to the microplate well, which was then left to incubate for 15 min at room temperature. Following incubation, 10 μL of DTNB (final concentration: 0.3 mM) and 10 mL of ATChI/BTChI (final concentration: 0.5 mM) were added to the reaction mixture. After four minutes, the absorbance was measured at 405 nm. Non-enzymatic hydrolysis was assessed as a blank for the control measurement in the absence of inhibitors and enzymes. The samples were tested using the non-enzymatic hydrolysis procedure with an additional inhibitor as a blank. The enzyme was swapped out for the same amount of buffer.

The inhibition % was determined using the following formula: Inhibition (%) = [(*A*_C_ − *A*_T_)/*A*_C_] × 100, where *A*_C_ is the activity of the enzyme without a test sample and *A*_T_ is the activity of the enzyme with a test sample. The mean values ± standard deviation are used to illustrate the results. Inhibitory activity of ethanol was deducted from each sample. A nonlinear fit of compound concentration values vs. response was used to determine the IC_50_ values. Each trazolinium salt was tested against both enzymes in triplicate.

### 3.4. Anti-Inflammatory Activity of ***1***–***17***

The effect of compounds on tumor necrosis factor alpha (TNFα) production in lipopolysaccharide (LPS)-stimulated peripheral blood mononuclear cells (PBMCs) was evaluated as described previously [25]. In short, PBMCs were isolated from buffy coats obtained from healthy adult volunteers and resuspended in RPMI1640 medium (Capricorn Scientific, Ebsdorfergrundu, Germany) supplemented with 10% heat-inactivated FBS (Biowest, Nuailléu, France), 1% GlutaMAX (Gibco, St. Louis, MO, USA), and 1% Antibiotic-Antimycotic (Gibco, St. Louis, MO, USA). PBMCs were seeded as 2 × 10^5^ per well of a 96-well plate. Test compounds were first dissolved in dimethyl sulfoxide (DMSO, Sigma, St. Louis, MO, USA) and three-fold serial dilutions were prepared in DMSO. Compounds were added to cells with a starting concentration of 100 µM. Cells were pre-incubated with compounds for 1 h and then stimulated with 1 ng/mL LPS from *E. coli* 0111:B4 (Sigma). Upon LPS stimulation, cells were incubated for 24 h at 37° C, 5% CO_2_, followed by the collection of supernatants for the measurement of TNFα and cell viability assessment.

For cell viability evaluation, CellTiter-Glo reagent was used (Promega, Radoboj, Croatia) according to the manufacturer’s instructions. Signals obtained in compound-treated cells were compared with signals in LPS-stimulated vehicle-treated samples. TNFα concentration in supernatants was measured by ELISA using antibodies and recombinant human TNFα protein (standard) from R&D Systems. Lumitrac 600 384-well plates (Greiner Bio-One, Kremsmünster, Austria) were coated overnight at 4 °C with 1 µg/mL of TNFα capture antibody diluted in phosphate-buffered saline (PBS; Gibco, St. Louis, MO, USA). The next day, plates were blocked for 4 h at RT with 5% sucrose (Kemika, Zagreb, Croatia) in assay diluent (1% bovine serum albumin (BSA; Sigma, St. Louis, MO, USA) in PBS). After the blocking step, samples and standard were added to plates followed by overnight incubation at 4 °C. The next day, 250 ng/mL of TNFα detection antibody was added to wells and incubated for 2 h at RT. Finally, after the plates were incubated with streptavidin HRP (Invitrogen, Thermo Fisher Scientific, Waltham, MA, USA), chemiluminescence ELISA Substrate (Roche, Zagreb, Croatia) was added to the wells and luminescence was measured using EnVision 2105 multilabel reader (Revvity, Waltham, MA, USA). Concentrations of TNFα in the supernatants were calculated using measured luminescence by interpolation from standard curves. Percentages of inhibition (PIN) were calculated from obtained cytokine concentrations and IC_50_ values were determined using GraphPad Prism v9 software using nonlinear regression curve fit (four parameters with variable slope).

## 4. Conclusions

In this study, a new series of thienobenzo-1,2,3-triazolinium bromide salts was synthesized and evaluated as potential dual cholinesterase inhibitors (AChE and BChE). The compounds were obtained using a photochemically assisted synthetic pathway, confirmed by structural analyses, and tested for biological activity. Emphasis was placed on BChE inhibition due to its increasing clinical relevance in the later stages of Alzheimer’s disease, where BChE expression is elevated in neurofibrillary tangles and amyloid plaques. Newly synthesized thiabenzo-1,2,3-triazolinium salts **1**–**4**, **7**–**11,** and **14**–**17** show potential as dual cholinesterase inhibitors, although most exhibit stronger inhibition of butyrylcholinesterase (BChE) compared to acetylcholinesterase (AChE). Compounds **8** and **11** stand out as the most potent AChE inhibitors, while compounds **1**, **2**, and **8**, with benzyl and methylbenzyl groups on the triazole ring, show very good and selective inhibition of BChE. This indicates further structural optimization is needed for strong AChE selectivity. Compound **17**, which showed the weakest cholinesterase inhibition, was the only one to exhibit mild anti-inflammatory activity by slightly suppressing TNF-α production in LPS-stimulated PBMCs. Most of the tested compounds showed no significant anti-inflammatory effects, suggesting that structural modification will be necessary to achieve multifunctional therapeutic profiles. A key contribution of this research is the demonstration that the electric charge of the triazolium core significantly influences cholinesterase inhibition. Additionally, the type and position of substituents play a critical role in determining selectivity and potency. Due to the selective and potent BChE inhibition shown by certain derivatives, these molecules hold promise as lead candidates in the development of new therapeutics for neurodegenerative disorders, especially Alzheimer’s disease, where BChE-targeting drugs are particularly relevant in the late stages. Future studies will be focused on optimizing anti-inflammatory properties, assessing pharmacokinetics and toxicology, and evaluating in vivo efficacy of the most active compounds.

## Figures and Tables

**Figure 4:**
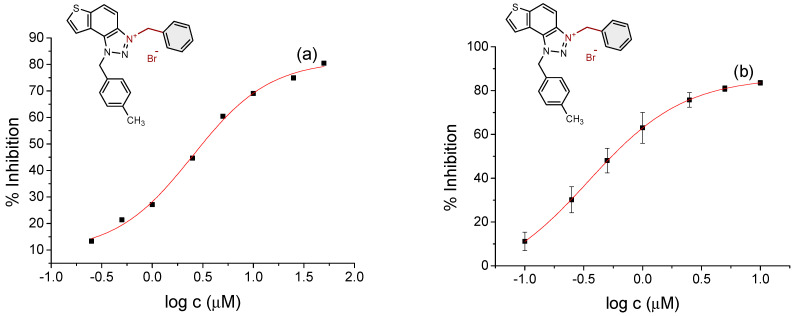
Dose–response curve for the inhibition of AChE (**a**) and BChE (**b**) by thienobenzo-1,2,3-triazolinium salt **8**.

**Figure 5:**
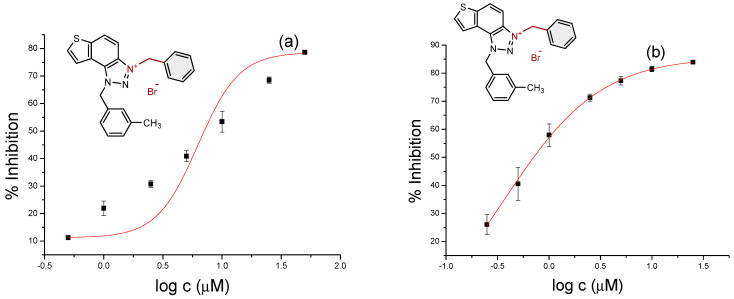
Dose-response curve for the inhibition of AChE (**a**) and BChE (**b**) by thienobenzo-1,2,3-triazolinium salt **1**.

**Table 1:** Cholinesterase inhibition activity of triazolinium salts **1**–**17**.

Compound	Structure	AChE		BChE	
		IC_50_/µM	Inhibition * (%)	IC_50_/µM	Inhibition * (%)
**1**	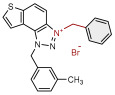	6.3	78.6 ± 0.5 (50)	0.4	83.9 ± 0.2 (25)
**2**	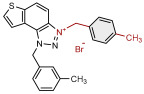	4.1	82.1 ± 4.2 (50)	0.3	77.6 ± 0.3 (2.5)
**3**	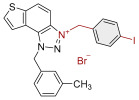	5.9	76.7 ± 0.5 (50)	0.7	84.9 ± 3.6 (50)
**4**	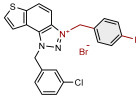	14.2	74.6 ± 5.0 (50)	0.8	83.2 ± 0.9 (25)
**7**	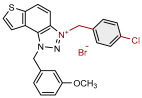	11.8	79.4 ± 4.9 (100)	1.0	78.2 ± 0.4 (10)
**8**	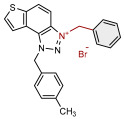	2.6	80.7 ± 2.3 (50)	0.4	83.6 ± 1.0 (10)
**9**	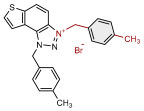	5.7	78.7 ± 0.6 (50)	3.5	76.6 ± 0.4 (25)
**10**	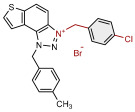	6.5	81.9 ± 0.6 (100)	1.5	79.9 ± 1.1 (25)
**11**	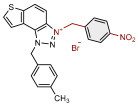	3.2	83.6 ± 2.1 (50)	2.7	81.1 ± 1.7 (25)
**14**	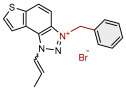	4.3	84.2 ± 0.5 (100)	1.0	80.4 ± 2.9 (25)
**15**	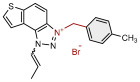	14.4	82.3 ± 0.8 (250)	0.9	84.0 ± 0.7 (50)
**16**	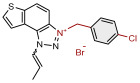	5.4	82.6 ± 1.1 (250)	2.3	84.8 ± 0.8 (100)
**17**	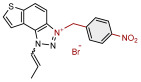	63.8	62.6 ± 5.3 (100)	11.4	85.5 ± 0.5 (100)
	Galantamine [29]	0.15	-	7.9	-

* The numbers in parentheses represent the maximum concentrations tested in µM.

## Data Availability

Dataset available on request from the authors.

## Supplementary Materials

The following supporting information can be downloaded at: https://www.mdpi.com/article/10.3390/molecules30163439/s1, Figures S1–S13: dose–response curves for the inhibition of AChE (a) and BChE (b) by **1**–**17**.
