# Supplementary material for: Photochemically Assisted Synthesis of Thienobenzotriazole-Based Dual Cholinesterase Inhibitors
Source: Molecules. 2025 Aug 20;30(16):3439. doi: 10.3390/molecules30163439 (PMC12388126; doi:10.3390/molecules30163439)
Supplement: Supplementary file 1 [file molecules-30-03439-s001.zip › molecules-3810044-supplementary.pdf]

molecules

Article

Photochemically-Assisted Synthesis of Thienobenzotriazole-Based Dual Cholinesterase Inhibitors

Antonija Jelčić [1], Stanislava Talić [2,*], Ilijana Odak [2], Paula Pongrac [3], Dora Štefok [3], Martina Bosnar [3] and Irena Škorić [1,*]

1 Department of Organic Chemistry, University of Zagreb Faculty of Chemical Engineering and Technology, Trg Marka Marulića 19, HR-10 000 Zagreb, Croatia
2 Department of Chemistry, Faculty of Science and Education, University of Mostar, Matice Hrvatske bb, 88 000 Mostar, Bosnia and Herzegovina
3 Faculty of Biotechnology and Drug Development, University of Rijeka, Radmile Matejčić 2, HR-51 000 Rijeka, Croatia
* Correspondence: stanislava.talic@fpmoz.sum.ba (S.T.); iskoric@fkit.unizg.hr (I.Š.)

Table of contents:

1) Dose-response curves for the inhibition of AChE (a) and BChE (b) by **1–17**.

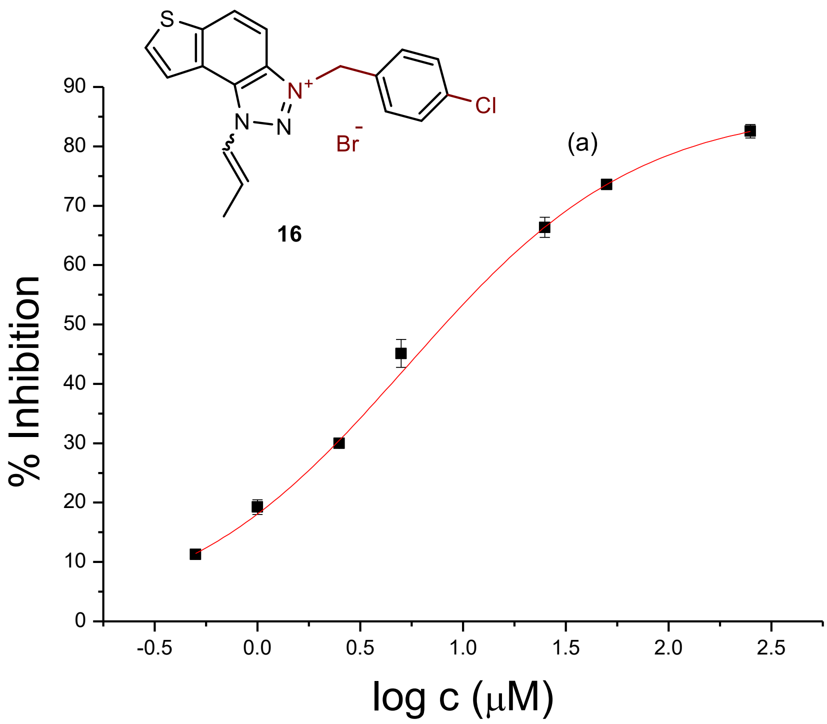

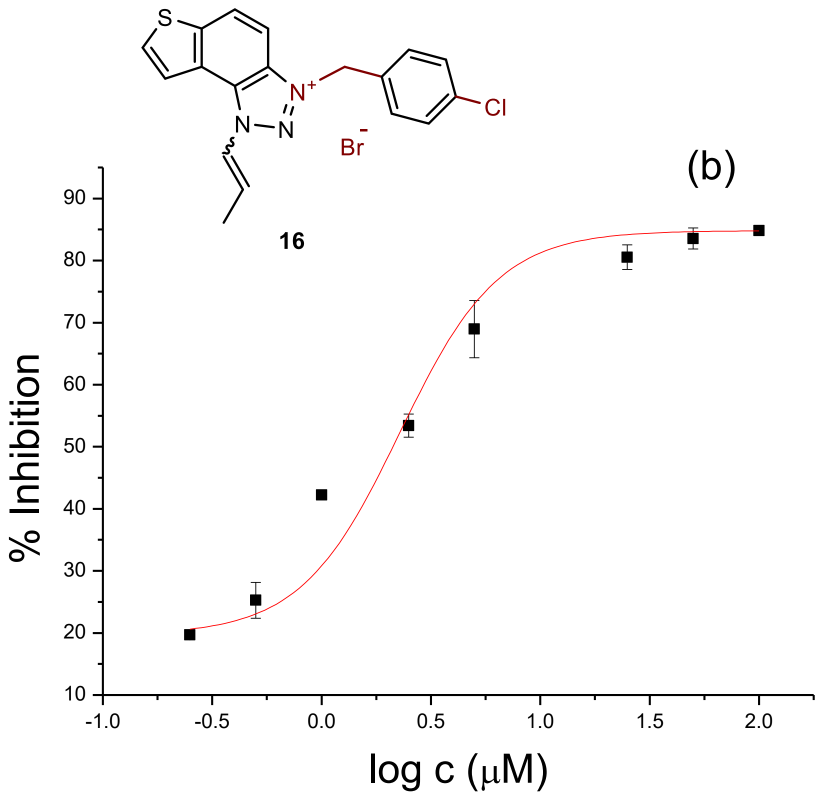

Figure S1. Dose-response curve for the inhibition of AChE (a) and BChE (b) by **16**.

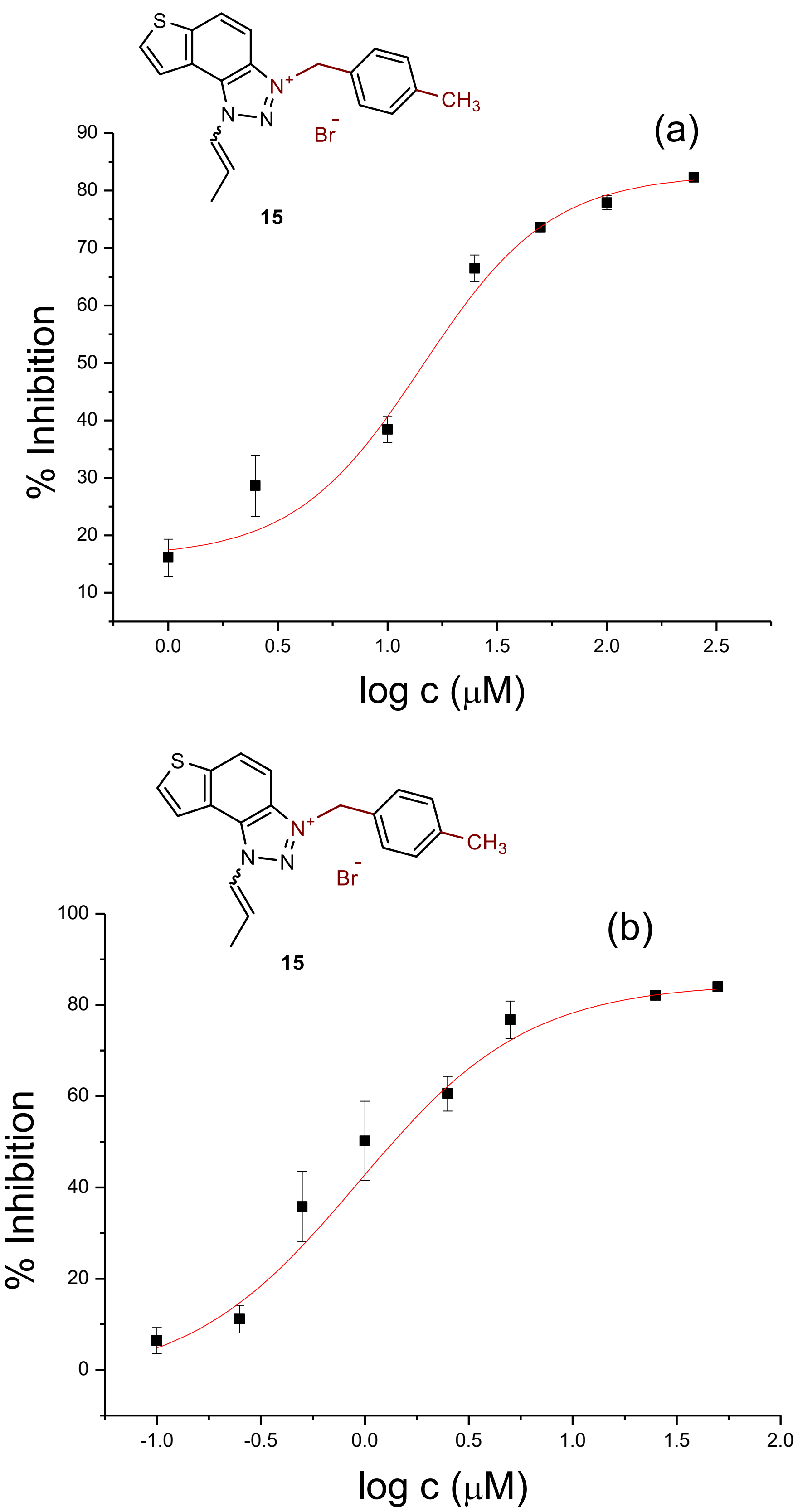

Figure S2. Dose-response curve for the inhibition of AChE (a) and BChE (b) by **15**.

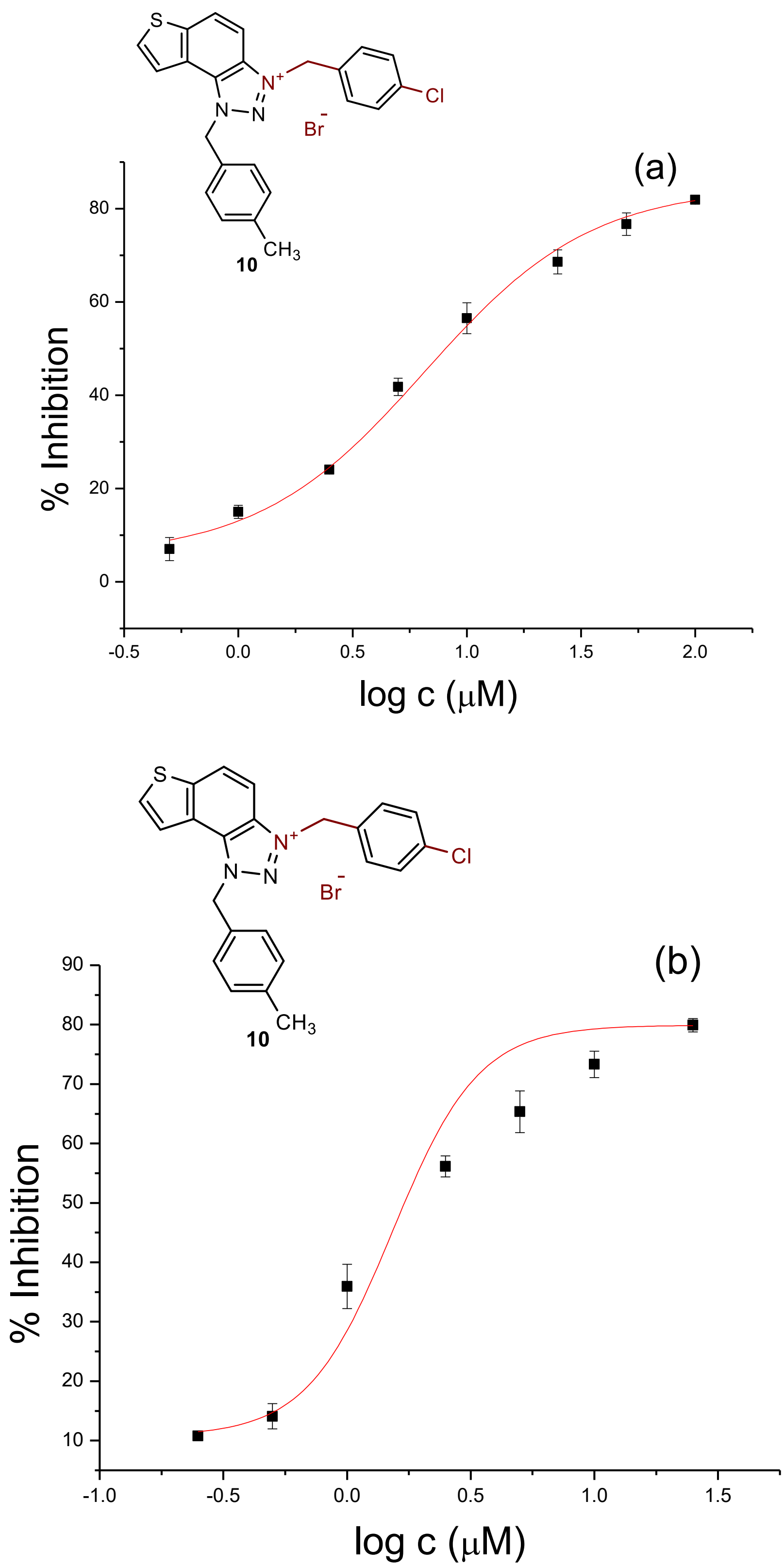

Figure S3. Dose-response curve for the inhibition of AChE (a) and BChE (b) by **10**.

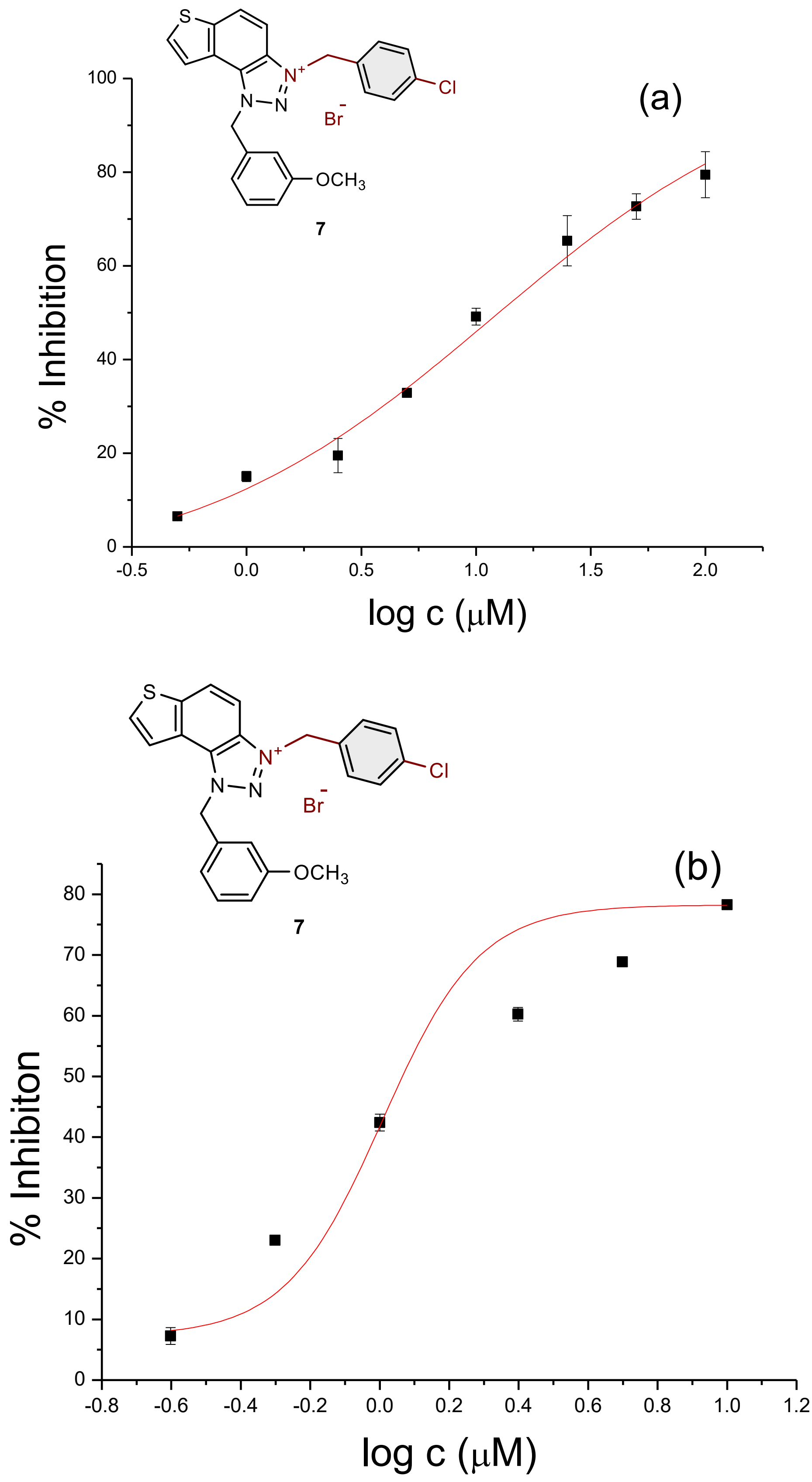

Figure S4. Dose-response curve for the inhibition of AChE (a) and BChE (b) by **7**.

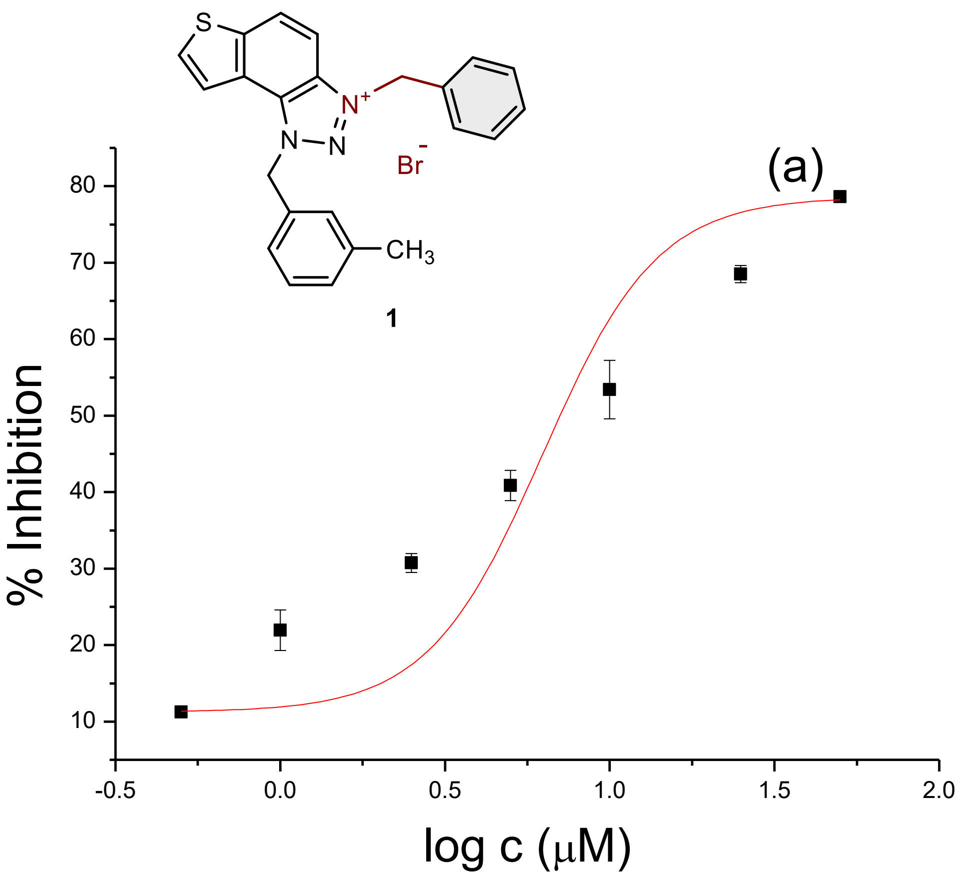

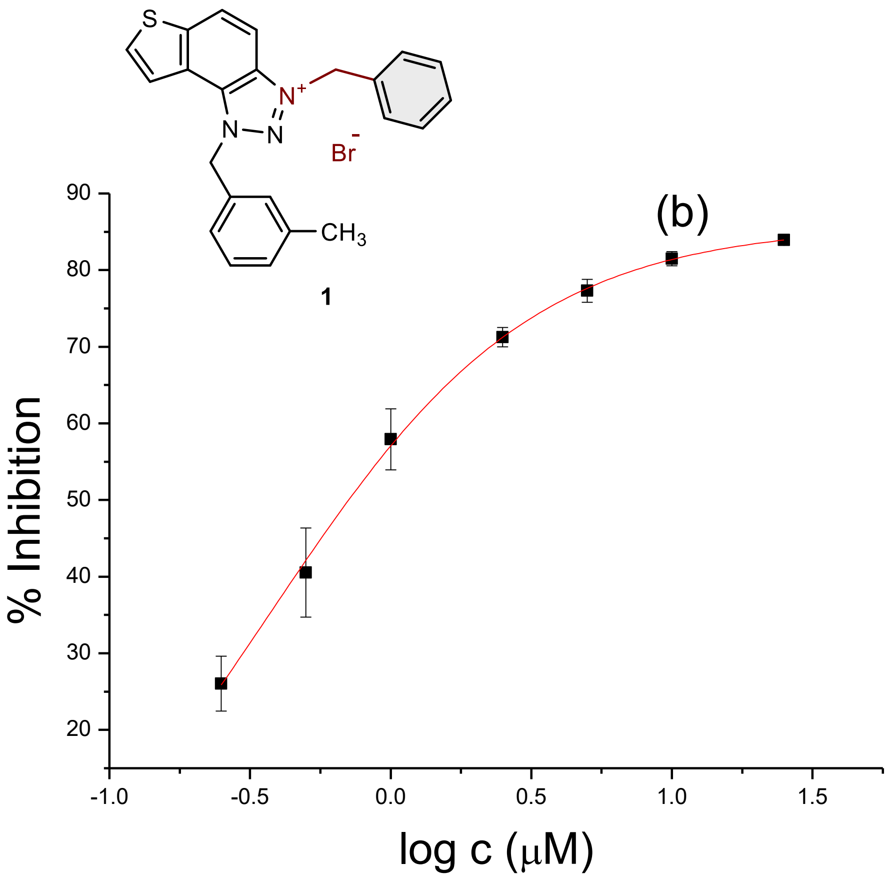

Figure S5. Dose-response curve for the inhibition of AChE (a) and BChE (b) by **1**.

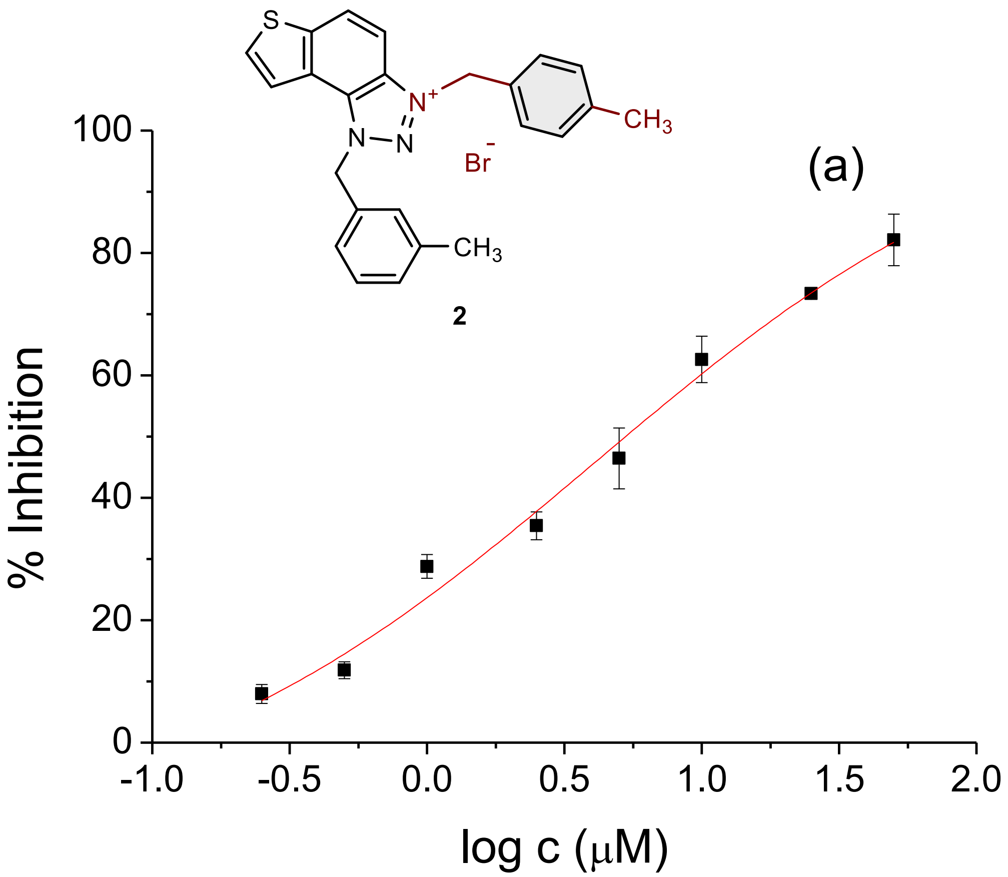

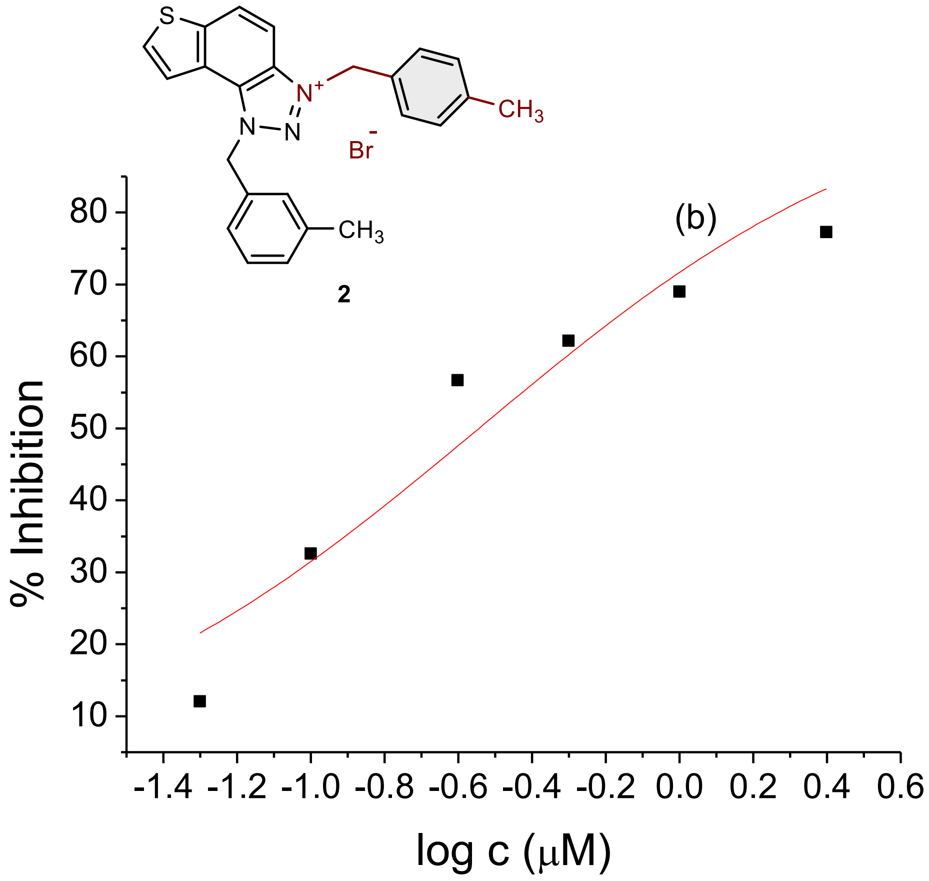

Figure S6. Dose-response curve for the inhibition of AChE (a) and BChE (b) by **2**.

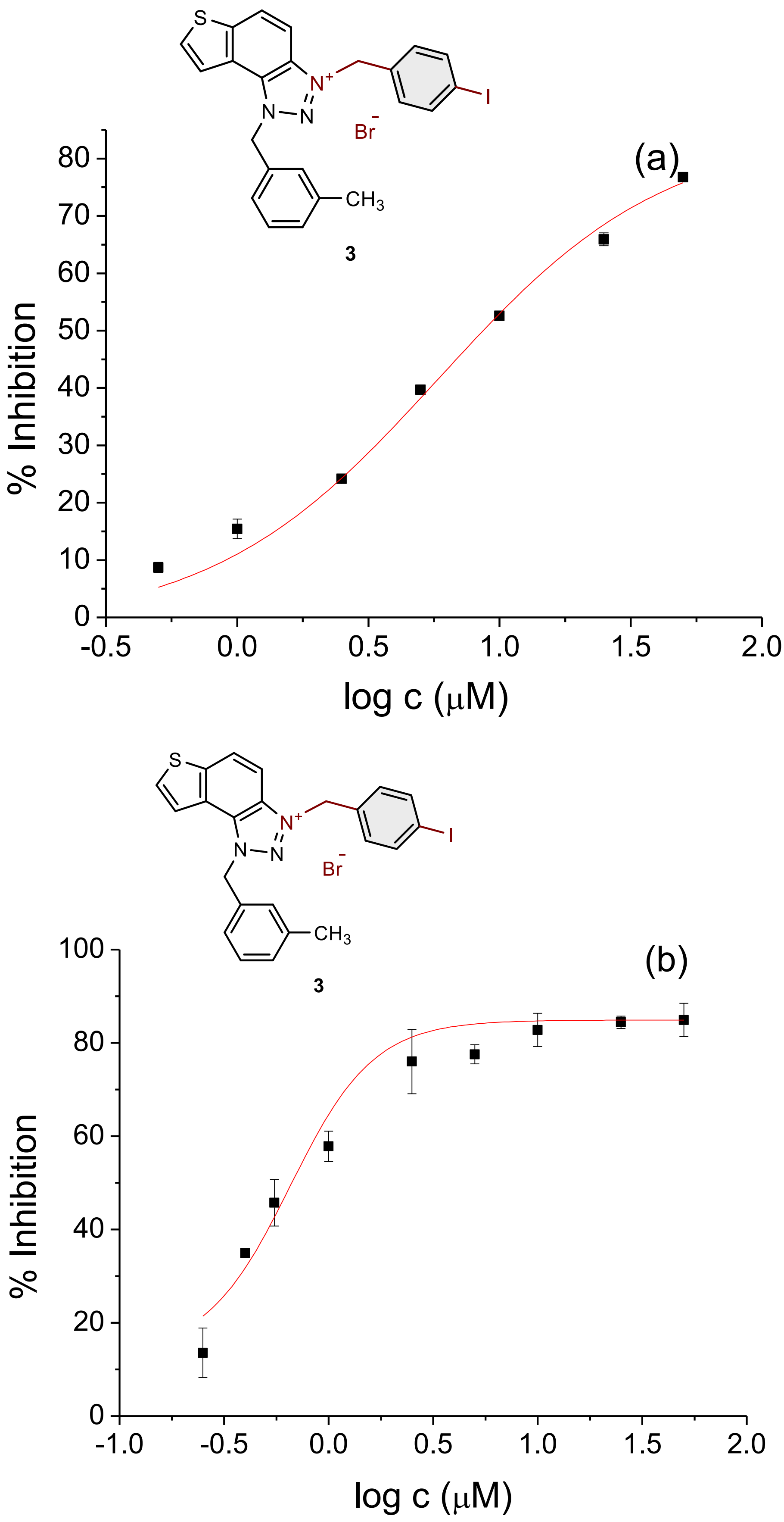

Figure S7. Dose-response curve for the inhibition of AChE (a) and BChE (b) by **3**.

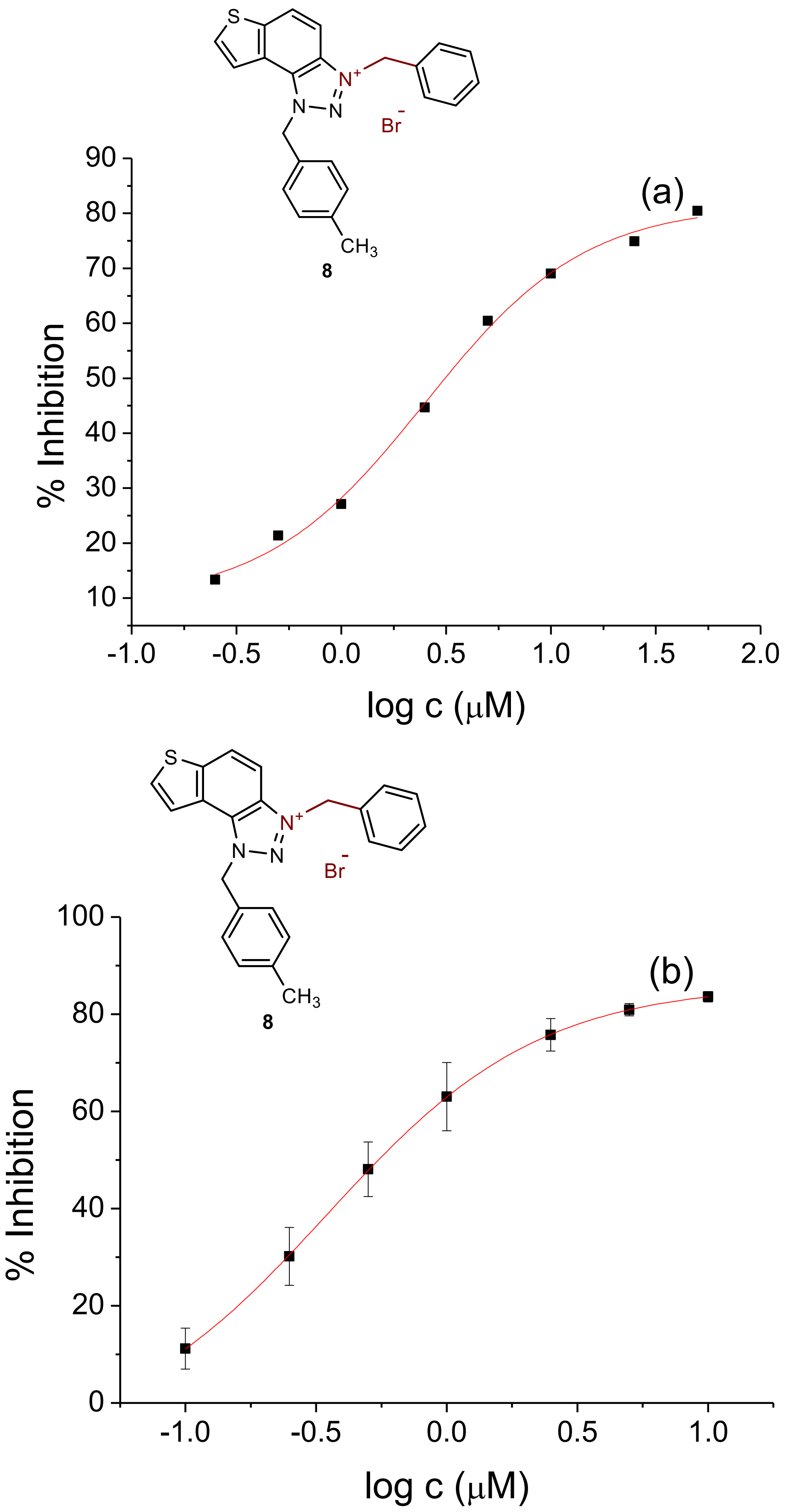

Figure S8. Dose-response curve for the inhibition of AChE (a) and BChE (b) by **8**.

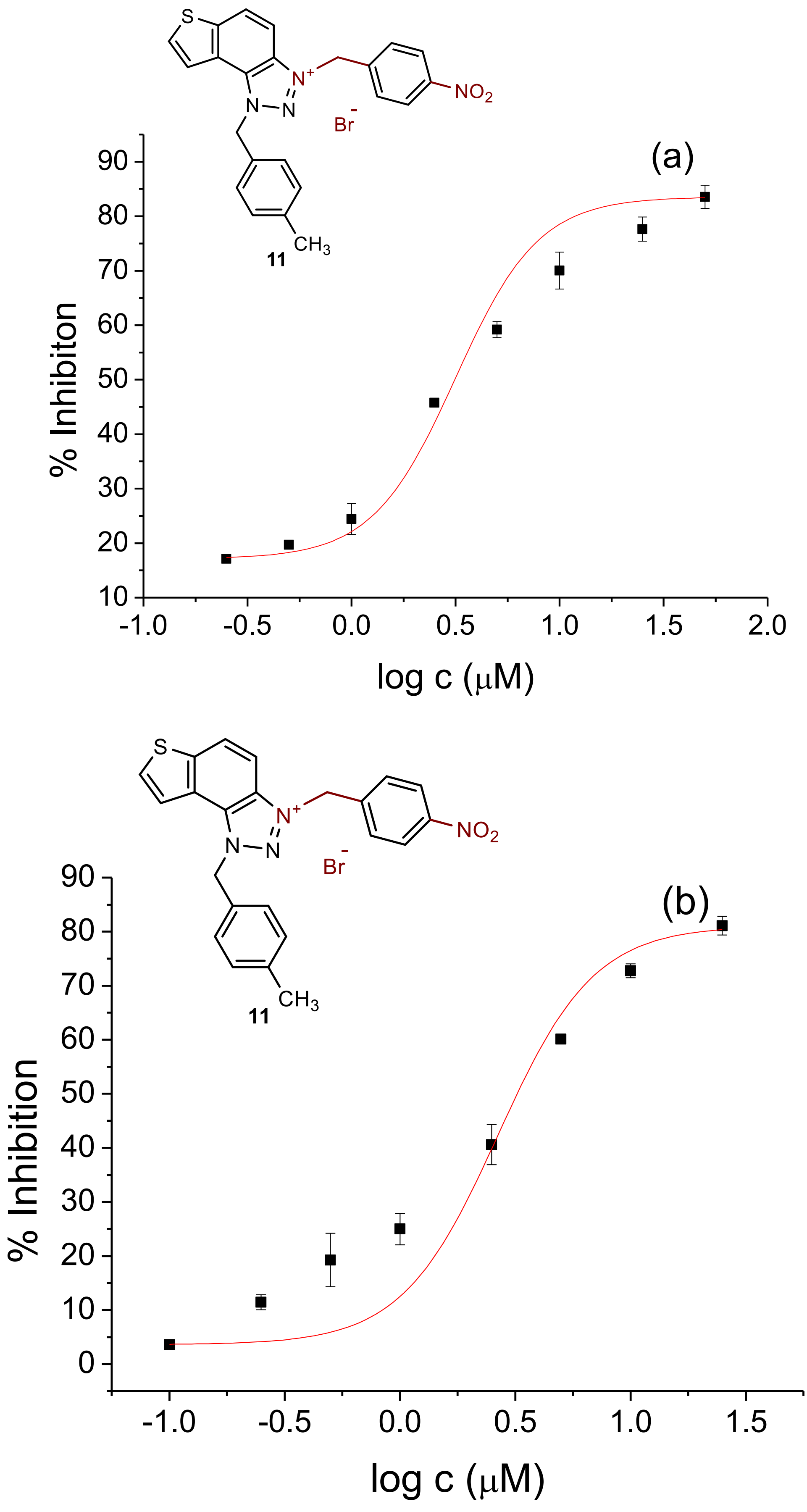

Figure S9. Dose-response curve for the inhibition of AChE (a) and BChE (b) by **11**.

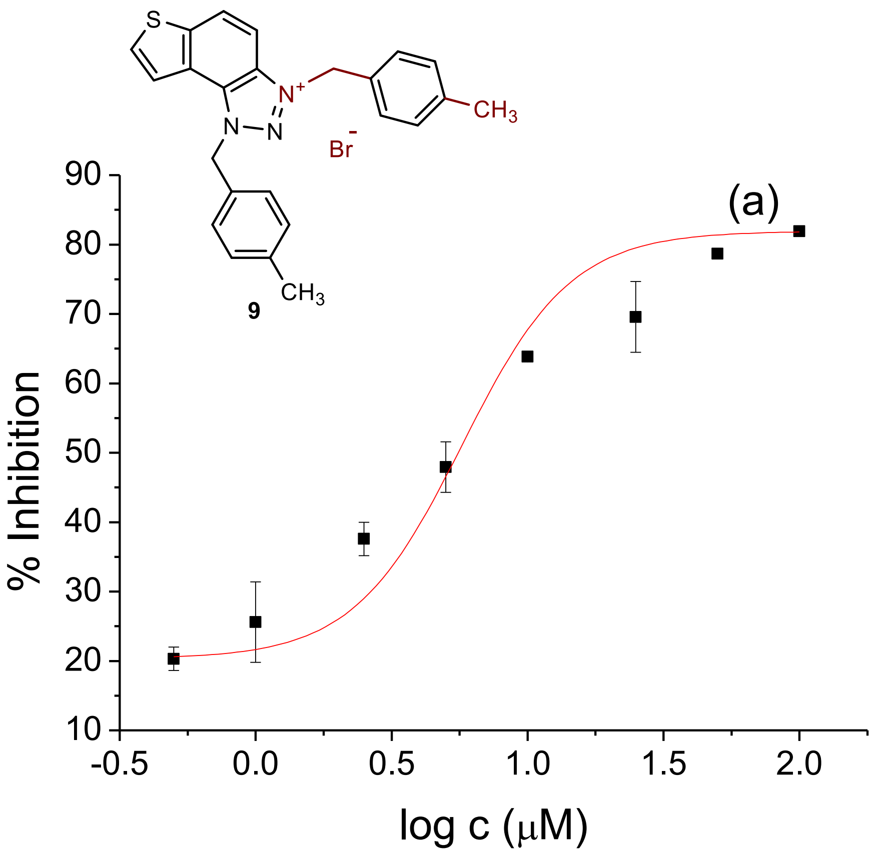

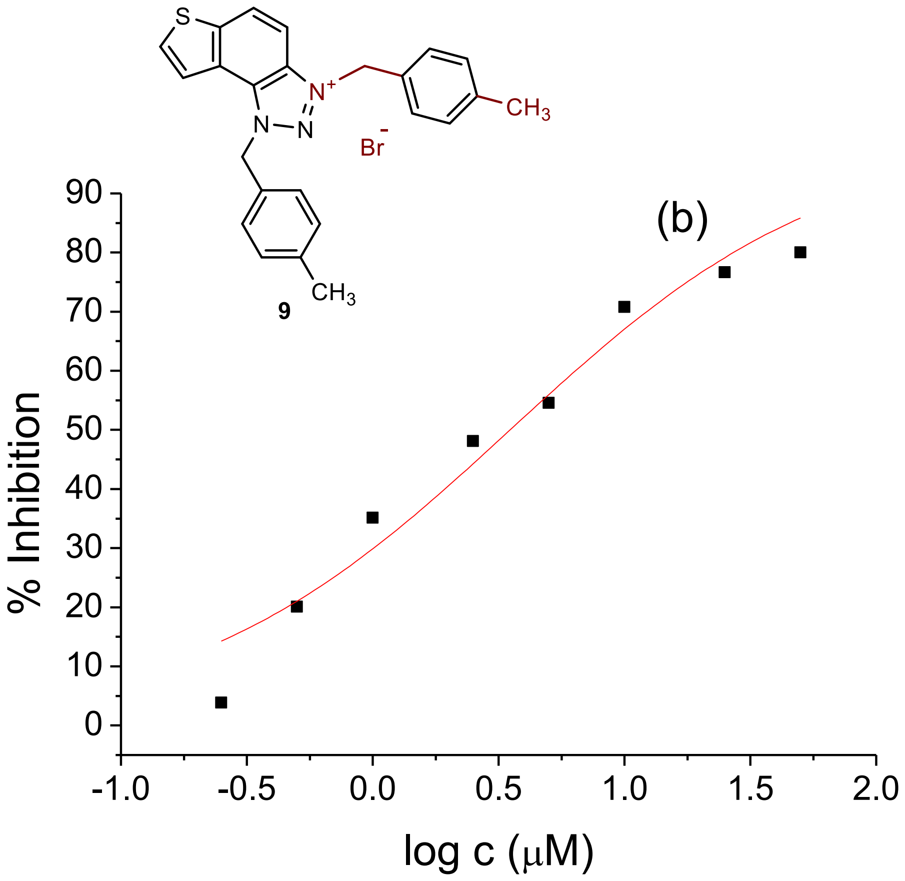

Figure S10. Dose-response curve for the inhibition of AChE (a) and BChE (b) by **9**.

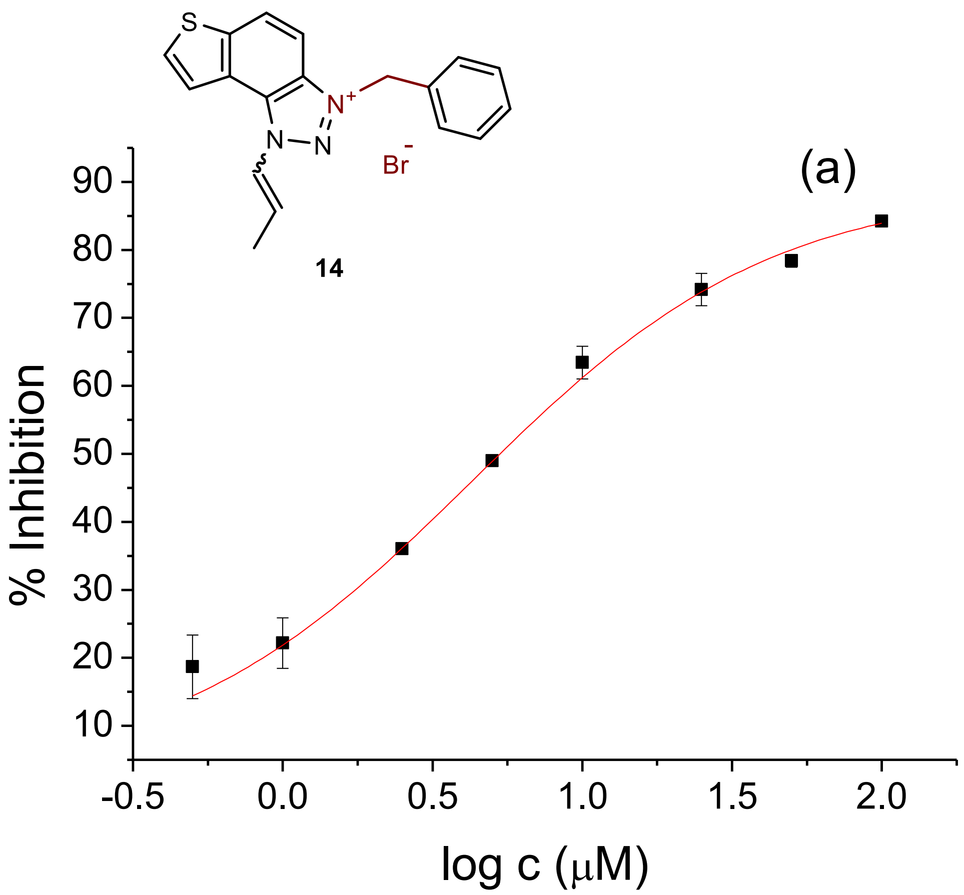

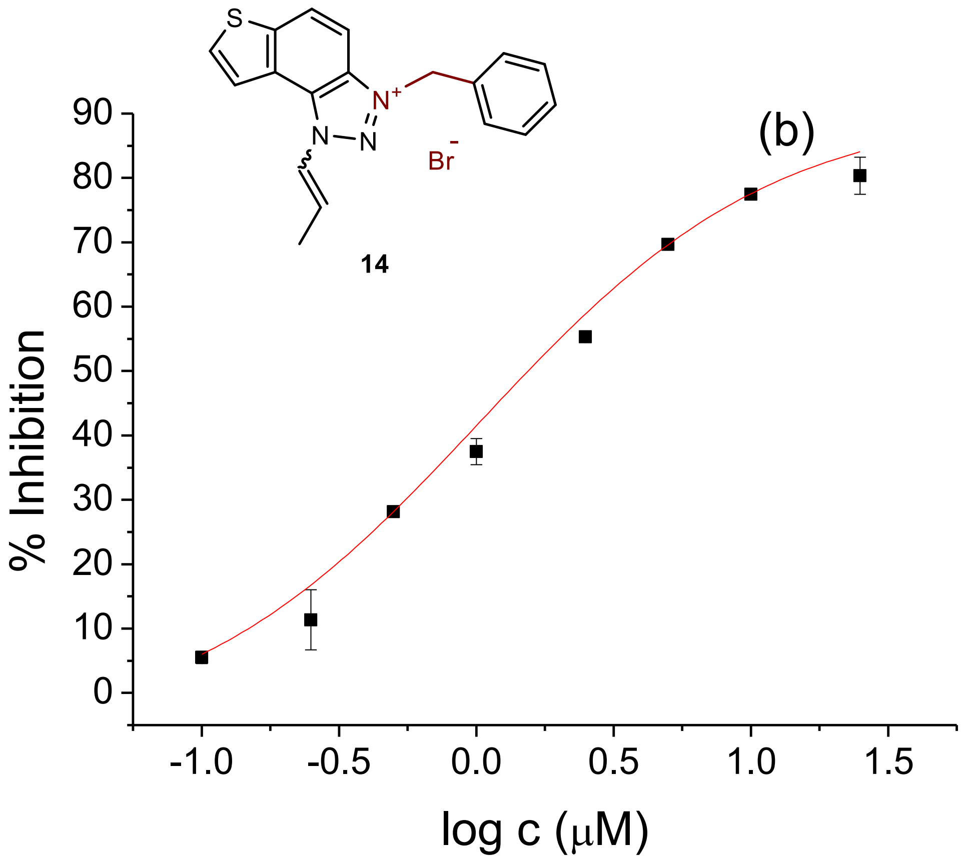

Figure S11. Dose-response curve for the inhibition of AChE (a) and BChE (b) by **14**.

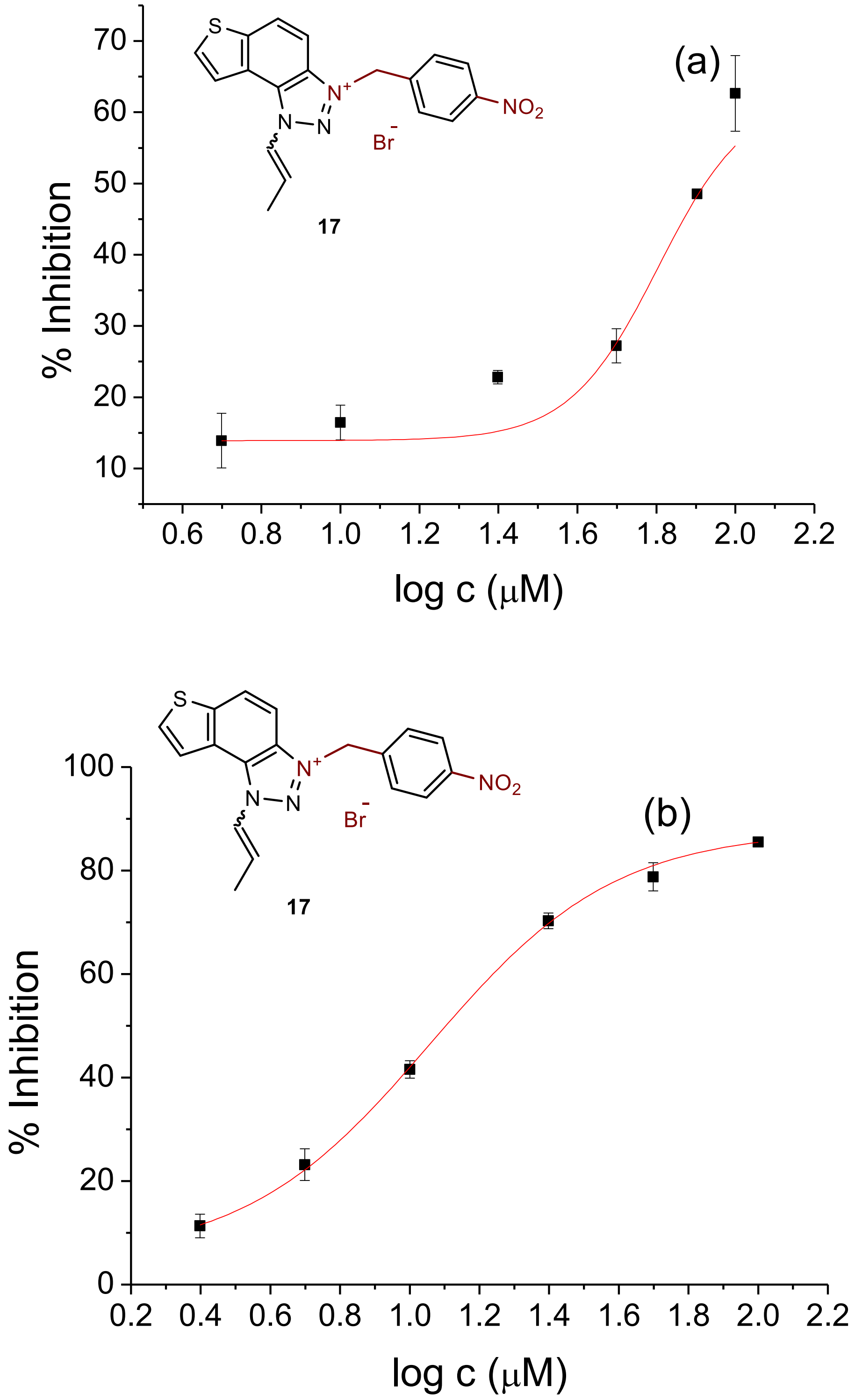

Figure S12. Dose-response curve for the inhibition of AChE (a) and BChE (b) by **17**.

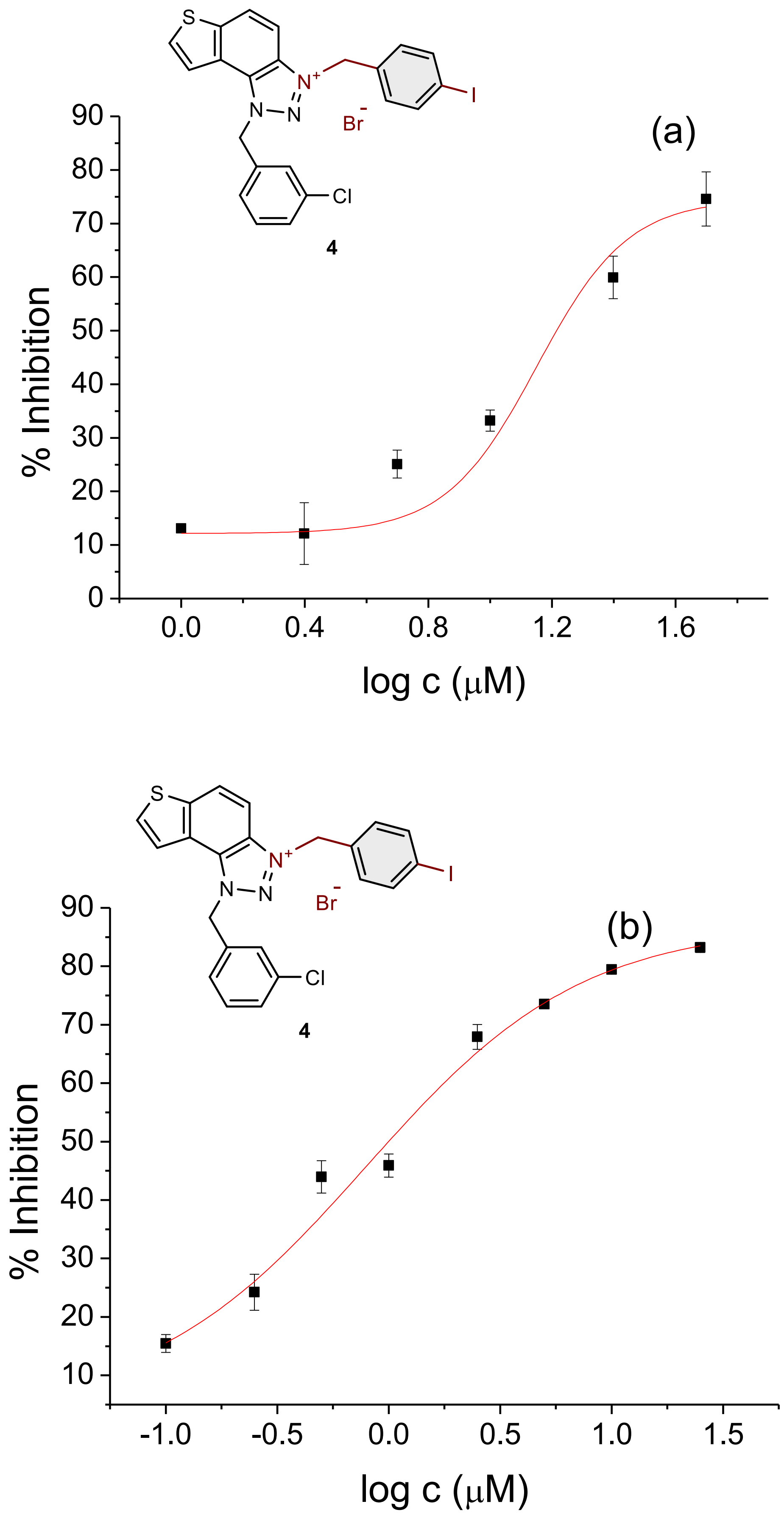

Figure S13. Dose-response curve for the inhibition of AChE (a) and BChE (b) by **4**.
